# Quantifying Induced Polarization of Conductive Inclusions in Porous Media and Implications for Geophysical Measurements

**DOI:** 10.1038/s41598-020-58390-z

**Published:** 2020-02-03

**Authors:** Lang Feng, Qiuzi Li, Stephen D. Cameron, Kuang He, Robert Colby, Katie M. Walker, Harry W. Deckman, Deniz Ertaş

**Affiliations:** 1 Corporate Strategic Research, ExxonMobil Research and Engineering, 1545 Route 22 East, Annandale, NJ 08801 USA; 2 Present Address: Sofinnova Partners, 7-11 Boulevard Haussmann, Paris, 75009 France

**Keywords:** Geophysics, Fossil fuels, Porous materials

## Abstract

Induced polarization (IP) mapping has gained increasing attention in the past decades, as electrical induced polarization has been shown to provide interesting signatures for detecting the presence of geological materials such as clay, ore, pyrite, and potentially, hydrocarbons. However, efforts to relate complex conductivities associated with IP to intrinsic physical properties of the corresponding materials have been largely empirical. Here we present a quantitative interpretation of induced polarization signatures from brine-filled rock formations with conductive inclusions and show that new opportunities in geophysical exploration and characterization could arise. Initially tested with model systems with solid conductive inclusions, this theory is then extended and experimentally tested with nanoporous conductors that are shown to have a distinctive spectral IP response. Several of the tests were conducted with nano-porous sulfides (pyrite) produced by sulfate-reducing bacteria grown in the lab in the presence of a hydrocarbon source, as well as with field samples from sapropel formations. Our discoveries and fundamental understanding of the electrode polarization mechanism with solid and porous conductive inclusions suggest a rigorous new approach in geophysical exploration for mineral deposits. Moreover, we show how induced polarization of biologically generated mineral deposits can yield a new paradigm for basin scale hydrocarbon exploration.

## Introduction

As a key electromagnetic-based geophysical exploration method^1–5^, induced polarization (IP) has been widely utilized in field-scale geophysical surveys for many decades^6–9^ to provide information about the complex conductivity (chargeability) of subsurface formations filled with ionically conducting fluids. These surveys supplement controlled-source electromagnetic^2^ and magnetotelluric^1,5^ surveys, which generate subsurface resistivity maps.

Frequencies of interest in these geophysical surveys are usually orders of magnitude below the Debye relaxation frequency (*ω*_*D*_) so ionic conduction dominates the electromagnetic response. There are two distinct, commonly recognized mechanisms that give rise to complex conductivity in this low-frequency regime^4^: (**1**) diffusive relaxation of neutral modes associated with ionic concentrations, usually called “membrane polarization”, and (**2**) capacitive charging of the electric double layer on the surfaces of non-ionic conductors, usually called “electrode polarization”. Regardless of the dominant mechanism, frequency dependence of complex conductivity is typically fitted to a Cole-Cole type model^10^ as the basis of various geophysical exploration methods^6–9,11–18^, where the effective complex conductivity of the porous media *σ*_*eff*_ has the form^16^:

$${\sigma }_{eff}(\omega )={\sigma }_{\infty }\left(1-\frac{m}{1+{(i\omega \tau )}^{c}\,}\right).$$

Here, *ω* is the angular frequency of the applied electric field and *σ*_∞_ is the high frequency limit of *σ*_*eff*_. The “chargeability” *m* quantifies the relative change in conductivity between the low- and high-frequency limits, whereas *τ* is a characteristic relaxation time and *c* is the Cole-Cole exponent. This model has originated from dielectric spectroscopy and has been successfully developed to understand metal-dielectric systems^19–21^, although its application to model observed IP response^16,22,23^ has been largely empirical.

Membrane polarization^4,9,12^ usually ties to IP effects observed in clay and shaley sand^3,9,24,25^, in glass materials such as silica sands with a Stern layer (also known as “charge polarization”^26,27^), and ion-selective membranes in electrochemical cells (also known as “concentration polarization”^28^). This type of polarization originates from inhomogeneities in the ionic transport properties, which drive ionic concentration gradients that ultimately relax through diffusion, therefore it leads to a relaxation time *τ* ∼ *a*^2^/*D*, where *a* is a characteristic length scale such as pore or grain size, and *D* is a characteristic diffusivity of dominant ions. In geophysical settings, this mechanism is more pronounced at lower pore fluid salinity (e.g., 1 mM NaCl or lower). Chargeability, which directly relates to the magnitude of the signal in IP surveys, diminishes at the higher salinities that are typically encountered below freshwater aquifers and in offshore marine settings.

In contrast, electrode polarization^9,13,24^ requires the presence of conductive grains with non-ionic charge carriers, for example electronic conductors or semiconductors, such as gold or sulfide ores like pyrite^23,29–32^. These grains act as short circuits in the high-frequency limit and insulators in the low-frequency limit, therefore the chargeability depends primarily on the effective volume fraction of the conductive grains, and does not diminish at higher salinities. Unlike membrane polarization, the electrode polarization relaxation time has strong dependence on the conductivity of the pore fluid^22,30,32^. There are several mechanistic and semi-empirical models for electrode polarization^29,31–33^ with broad agreement on chargeability but very different explanations for the relaxation time.

In this study, we show that capacitive charging of the Stern layer on the conductive grain is the primary mechanism for the observed electrode polarization relaxation time. This mechanism imparts a distinct and quantifiable spectral IP response linking directly to the intrinsic physical properties of the associated rocks, such as feature size, surface area, and electrical properties of the grains. In high salinity environments this mechanism dominates the observed IP signal and enables us to relate the response to the intrinsic physical properties of the conductive particulates. It also enables us to extend this mechanistic model to porous conducting grains in order to demonstrate the profound influence of nanoporous conductors on the characteristic relaxation time of the IP signal. We then discuss the geophysical implications of this insight in various geological settings. Treatment of IP response when membrane polarization effects are significant is beyond the scope of this study. Typically this occurs in low salinity environments.

### Induced polarization with solid conductive inclusions

**Figure 1:**
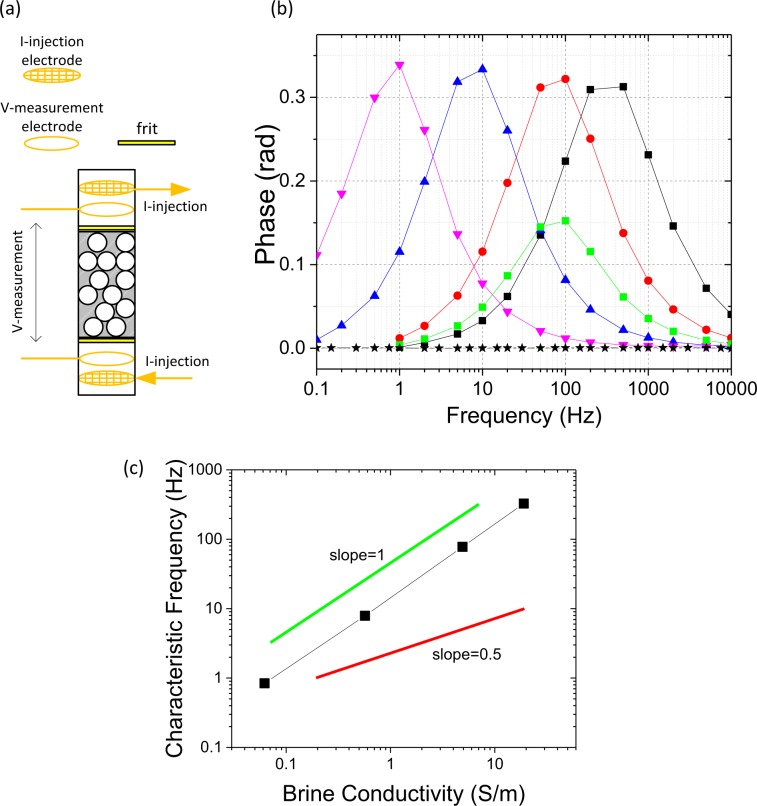
Induced polarization in porous media with conductive inclusions, (**a**) Graphic view of four-probe experimental cell: current electrodes are meshes; voltage electrodes are rings; thin porous frits are used to hold glass bead pack in position. (**b**) Frequency dependent phase shift of glass bead pack with 17 mm cubic pyrites. Black stars: control sample with 3 wt% NaCl; Green Squares: 1 pyrite cube with 3 wt% NaCl; Red circles: 2 pyrite cubes with 3 wt% NaCl; Black Squares: 2 pyrite cubes with 15 wt% NaCl; Blue Triangles: 2 pyrite cubes with 0.3 wt% NaCl; Pink Inverted Triangles: 2 pyrite cubes with 0.03 wt% NaCl; (**c**) Characteristic frequencies versus brine conductivities (NaCl solution) for the samples containing 2 pyrite cubes.

To isolate the fundamental mechanism for the electrode polarization relaxation time, we performed a series of frequency domain experiments with model systems. One of our model systems consists of natural cubic pyrite crystals (each side ~17 mm) embedded in a framework of glass beads (average particle diameter ~0.4 mm). The model systems are held in a custom-designed four-probe measurement cell as shown in Figs. 1(a) and S2(a). We use a National Instruments system to generate sinusoidal waves and measure the response from the voltage electrodes, and obtained the phase shifts between the injected current and measured voltage in a frequency range from 0.1 Hz to 10000 Hz, with an uncertainty about 0.5mrad. (See Methods).

As controls, we run tests with either brine only or brine saturated glass bead packs with different salt concentrations. We observe no significant phase shifts in this frequency range (an example shown as stars in Fig. 1(b)), consistent with the literature result^26^ for salinities of 0.03 wt% NaCl or higher. With two cubic pyrite particles in the framework we observe significant frequency-dependent phase shifts in samples with different brine conductivities (with 0.03%, 0.3%, 3% and 15 wt% NaCl) in Fig. 1(b). We fit the complex conductivity over the entire frequency range to an effective RC circuit shown in Fig. S2(b), and find that the maximum phase shift *ϕ*_*c*_ shows no discernible dependence on the brine conductivity *σ*_*w*_ (Figs. 1(b) and S3), while the characteristic frequency *f*_*c*_ at the peak depends on brine conductivity almost linearly (Fig. 1(c)). This result suggests that the electrolytic capacitance associated with the Stern layer at the conductor-electrolyte interface is the primary origin of complex conductivity in this metal-electrolyte system (See SI-Text-S1).

Based on this experimental insight, we constructed a theoretical model to derive the effective complex conductivity *σ*_*eff*_ and subsequently the polarization effects associated with solid electronically conductive spheres of radius a with surface specific capacitance^34–36^ *C*_0_ dispersed in brine-filled porous media. *σ*_*eff*_ is found to be (See SI-Text-S1 for detailed derivation):

1 $${\sigma }_{eff}={\sigma }_{m}\left[1+3{V}_{cond}\left(1-\frac{3}{2}\frac{1}{1+if/{f}_{c}\,}\right)\right]\,{\rm{with}}\,{\rm{peak}}\,{\rm{frequency}}\,{f}_{c}=\frac{{\sigma }_{m}}{\pi a{C}_{0}}.$$

Here the porous media (without conductive grains) is described in terms of its effective conductivity *σ*_*m*_, the brine conductivity *σ*_*w*_ and the corresponding formation factor F = *σ*_*w*_/*σ*_*m*_. *V*_*cond*_ is the volume fraction of the conductors and is much less than one. Equation (1) corresponds to a Cole-Cole Model with exponent *c* = 1, while now the chargeability *m* = 9*V*_*cond*_/2(1+3*V*_*cond*_) and relaxation time *τ* = *aC*_*0*_/2*σ*_*m*_ link directly to intrinsic properties of the porous media. Equation(1) makes two explicit predictions: (**1**) the maximum phase shift ${\phi }_{c}=\frac{9}{4}\frac{{V}_{cond}}{1+3{V}_{cond}}$ only depends on volume fraction of the conductive inclusions, and (**2**) *ϕ*_*c*_ occurs at the characteristic frequency ${f}_{c}=\frac{{\sigma }_{m}}{\pi a{C}_{0}}$ that scales linearly with brine conductivity, and also depends on *a* and *C*_*0*_.

**Figure 2:**
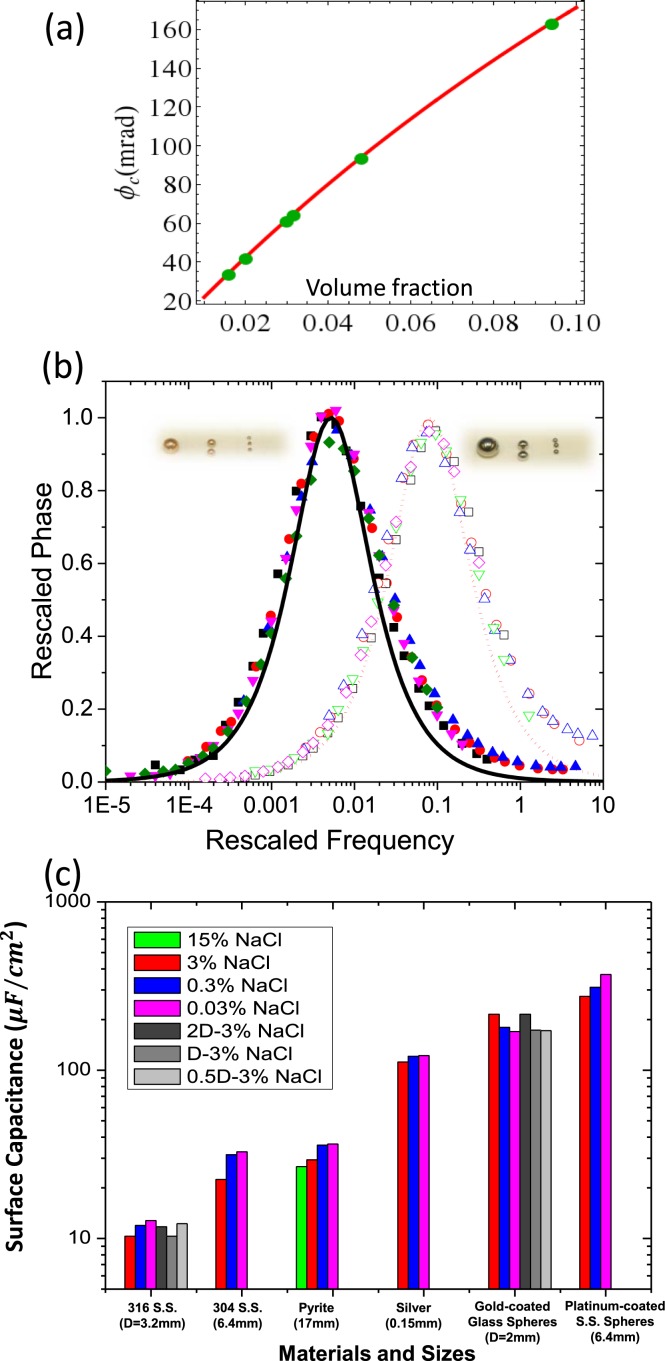
Experimental measurements and surface capacitances for different metals. (**a**) Maximum phase shifts versus volume fraction of conductive inclusions (e.g., gold-coated glass spheres, silver grain, stainless steel and platinum coated stainless steel). Solid red curve is theoretical prediction with no fitting parameter. (**b**) Conductivity phase spectra with rescaled frequency ${f}^{\ast }=\frac{\pi f\,a\,{C}_{unit}}{{\sigma }_{m}}$ and phase shift ${\phi }^{\ast }=\phi \frac{4(1+3{V}_{cond})}{9{V}_{cond}}$. Solid symbols present the rescaled data with gold-coated glass sphere inclusions: 7.6% v/v 4 mm gold-coated glass spheres with 0.03 wt% NaCl (Blue triangles), 0.3 wt% NaCl (Red circles), and 3 wt% NaCl (Black squares); Pink inverted triangles: 7.0% v/v 2 mm gold-coated glass spheres with 3 wt% NaCl; Green diamonds: 4.9% v/v 4 mm gold-coated glass spheres with 3 wt% NaCl. Open symbols present the rescaled data with 316 stainless steel spheres: 3.2% v/v 3.2 mm spheres with 0.03 wt% NaCl (Blue triangles), 0.3 wt% NaCl (Red circles) and 3%wt NaCl (Black squares); Green inverted triangles: 3.2% v/v 6.4 mm spheres with 3 wt% NaCl; Pink diamonds: 3.5% v/v 1.6 mm spheres with 3 wt% NaCl. The solid black line and dotted red line are our theoretical spectral curves after rescaling with characteristic frequencies ${f}_{c}^{\ast }\, \sim \,0.005$ (gold-coated glass spheres) and ${f}_{c}^{\ast }\, \sim \,0.08$ (316 stainless steel spheres). Here the characteristic frequency is the single fitting parameter for each set of curves. (**c**) Surface specific capacitance measurements for various conductors, guided by theoretical analysis.

To validate our theoretical predictions, we tested gold-coated glass spheres, silver grains, 304 and 316 stainless steels, and platinum-coated stainless steels of different sizes across different conditions (see Methods and SI-Table-S1). For all samples, measurements of *ϕ*_*c*_ strongly agrees with the theoretical model ${\phi }_{c}=\frac{9}{4}\frac{{V}_{cond}}{1+3{V}_{cond}}$ with no fitting parameter as shown in Fig. 2(a). In Figs. 2(b) and S4, we show some examples with gold-coated glass spheres and 316 stainless steel spheres of different diameters, volume fractions and varying NaCl concentrations. Based on the theoretical relations, we can rescale phases to ${\phi }^{\ast }=\phi \frac{4(1+3{V}_{cond})}{9{V}_{cond}}$ and frequencies to a unitless ${f}^{\ast }=\frac{\pi f\,a\,{C}_{unit}}{{\sigma }_{m}}$ using the experimentally determined *V*_*cond*_, *a*, *σ*_*m*_ and a unit capacitance *C*_*unit*_ =1 *μ*F/cm^2^. In principle, each rescaled spectrum should have a maximum phase shift ${\phi }_{c}^{\ast }={\phi }_{c}\frac{4(1+3{V}_{cond})}{9{V}_{cond}}=1$ and a rescaled characteristic frequency ${f}_{c}^{\ast }=\frac{\pi {f}_{c}\,a\,{C}_{unit}}{{\sigma }_{m}}=\frac{{C}_{unit}}{{C}_{0}}$ that only depends on the intrinsic surface property of the conductor *C*_0_ and is an invariant to *a*, *σ*_*m*_, and *V*_*cond*_. The solid symbols in Fig. 2(b) show an excellent collapse of all rescaled data with gold-coated glass spheres, on a scaling curve derived from Eq. (1) shown as a solid black line with ${f}_{c}^{\ast } \sim 0.005$. Similarly the open symbols in Fig. 2(b) represent rescaled data with stainless steel spheres on a scaling curve derived from Eq. (1) shown as a dotted red line with ${f}_{c}^{\ast } \sim 0.08$. ${\phi }_{c}^{\ast }\approx 1$ for all curves.

The collapse of both gold-coated glass sphere and stainless steel sphere data onto the theoretically predicted frequency dependence indicate that our model is quantitative once the corresponding *C*_0_ of the conductive material is known. In electrochemistry, differential surface capacitance of metals is usually measured and modeled with different approaches^35,37–40^, but unlike the conventional electrochemistry experiments, our metal inclusions are not connected to external circuitry and act as floating capacitors with an unknown DC bias voltage dictated by their work function, and possibly their residual electronic charge. As a result, it is necessary to obtain the values of *C*_0_ in this setting for geophysical applications, and the relationship ${C}_{0}=\frac{1\,\mu {\rm{F}}/{{\rm{cm}}}^{2}}{{f}_{c}^{\ast }}$ provides a convenient way to measure *C*_0_. For gold-coated glass spheres, we obtain *C*_0_ ∼ 200 *μ*F/cm^2^, and for 316 stainless steel spheres, *C*_0_ ∼ 12 *μ*F/cm^2^ with minor dependence on brine composition. In Fig. 2(c), we show the measured *C*_0_ for a collection of conductive inclusions, from platinum, gold and silver, to stainless steel. We expect noble metals such as platinum, gold and silver to have high *C*_0_ owing to a naturally inert surface, whereas the passivation of chromium oxide and other oxide layers lead to the low *C*_0_ for pyrite (iron disulfide) and stainless steel, due to the greater effective distance between the electronic charge and the ionic charge in the Stern layer^34^.

### Induced polarization of porous conductive inclusions

This mechanistic understanding of the origin of electrode polarization leads to a testable theoretical prediction of the profound influence of nanoporous conductors on the IP signal in geophysical settings. Our experiments show that centimeter-scale grains of disseminated euhedral cubic pyrite of hydrothermal origin should exhibit short relaxation times of a few milliseconds (e.g., Fig. S5) in the subsea environment. This is contrasted with an observed 0.5–5 seconds relaxation time or *f*_*c*_ ∼ 1 Hz, attributed to presence of pyrites associated with hydrocarbon seepage above a known hydrocarbon reservoir^7^. Based on our quantitative model, generating this signature with solid (nonporous) pyrite would require the presence of meter-sized solid pyrite grains (with *σ*_*m*_ ∼ 0.2S/m and C_0_ ∼ 30 *μ*F/cm^2^). This morphology is inconsistent with this geological setting^7^ and is only known to occur in massive ore deposits created by magmatic or hydrothermal processes. In this geological setting with sedimentary pyrite formation above a hydrocarbon reservoir, typical grain sizes tend to be in the tens to hundreds of micrometers. Here we propose that in such geological settings, one possible way of achieving dramatically increased relaxation times with this mechanism is to consider morphologies with very high surface-area-to-volume ratios, such as nanoporous conductive grains (e.g., framboidal pyrite and nano-porous pyrite^41–43^). For porous conductive inclusions, Eq. (1) is not sufficient to describe the charging and discharging process: Ion transport and capacitive charging occurs throughout the brine-filled pore space within the porous conductor, leading to new physical scaling (See full derivations in SI-Text-S2). The characteristic frequency now scales as:

2 $${f}_{c} \sim \frac{{\sigma }_{m}}{\pi {C}_{0}{a}^{2}}\frac{3}{{s}_{f}}$$

**Figure 3:**
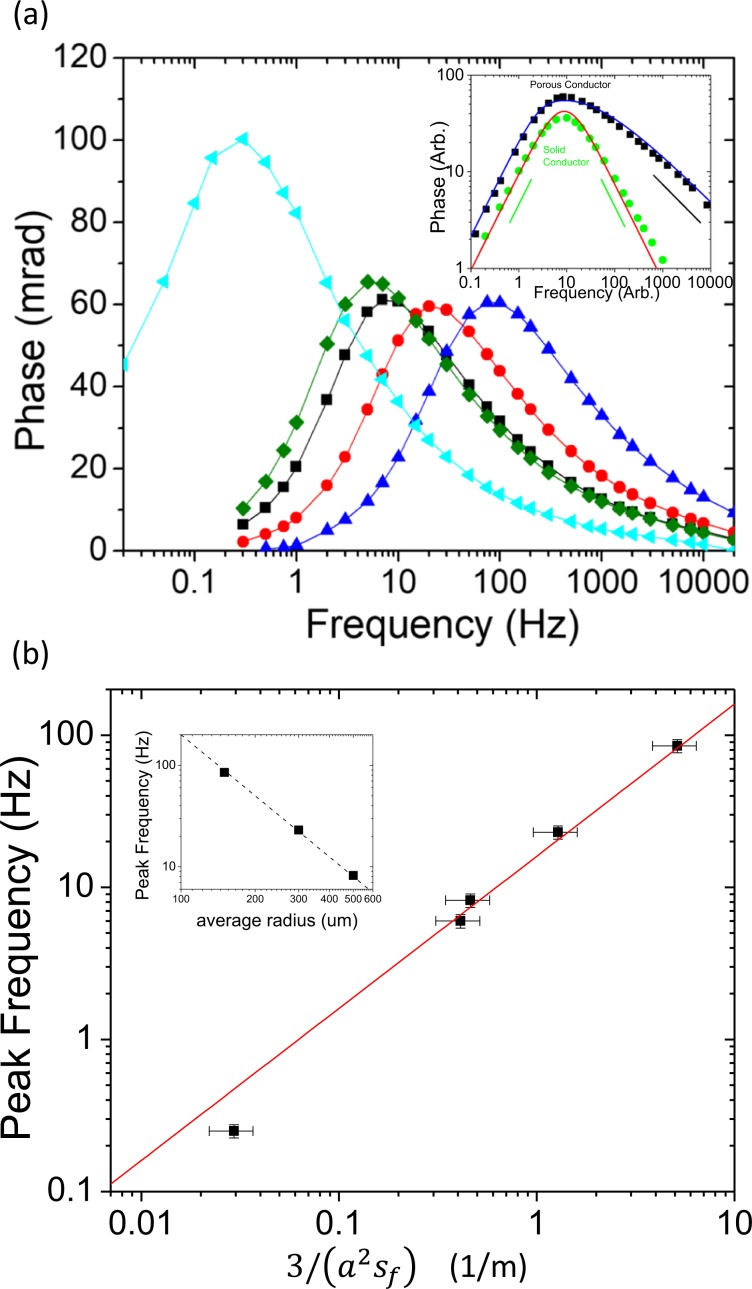
Induced Polarization of model porous conducting inclusions. (**a**) Induced polarization spectrum for a variety of porous carbon beads. Black, red and blue correspond to Black Pearl 120 of average radius 0.5 mm, 0.3 mm and 0.15 mm respectively. Green curve corresponds to Regal 660 of average radius 0.3 mm. Light blue curve corresponds to Darco 12-20 of average radius 0.5 mm. Inset: Phase vs. frequency plot in log-log scale for solid conductor (Fig. 2(b) rescaled) and porous conductor (**a**). Black squares and green circles correspond to experimental data of porous and solid conductors respectively. The solid red curve corresponds to a solid sphere model (Eq. 1), whereas the solid blue curve corresponds to a porous packing of spheres model (see Supplementary Information, Equations S3 and S4) with formation factor *F*_*f*_ ≈ 3. Left and right solid green lines are guide for scalings *ϕ* ∝ *f* ($f\ll {f}_{c}$) and *ϕ* ∝1/*f* ($f\gg {f}_{c}$). The black solid line is guide for scaling $\phi \propto 1/\sqrt{f}$ that only happens in porous conductor at high frequency ($f\gg {f}_{c}$). (**b**) Peak frequency *f*_*c*_ as a function of $\frac{3}{({a}^{2}{s}_{f})}$ for all data with porous carbon (black squares). Red line shows the theoretical curve ${f}_{c} \sim \frac{{\sigma }_{m}}{\pi {C}_{0}}\frac{3}{{a}^{2}{s}_{f}}$ with a fitted surface capacitance *C*_*0*_ ∼ 3 *μ*F/cm^2^. Inset: for Black Pearl 120 with same porosity parameter *s*_*f*_ and different size *a*, the peak frequency follows *f* ∝ 1/*a*^2^ almost exactly.

Here *s*_*f*_ stands for the surface-area-to-volume ratio of the specific porous conductors. (See Methods for detailed formulations of *s*_*f*_ for different porous conductors). To validate this modified scaling relation, we performed systematic experimental tests with a variety of nano-porous carbon beads as model materials. Carbon materials tested here are about a factor of 10^3^ more conductive than the 3 wt% NaCl solution used, and thus behave like a conductive inclusion. Furthermore, the surface-area-per-mass (*s*_*m*_) of commercially available porous carbon are often calibrated and benchmarked^44–46^, and *s*_*f*_ is a simple function of *s*_*m*_, *ρ* (density) and *φ* (porosity) as *s*_*f*_ = *s*_*m*_*ρ* (1−*φ*). In Fig. 3(a), we first tested Black Pearl 120 with *s*_*m*_ ∼ 25 m^2^/g^46^, sieved into different size ranges. In this case with a constant *s*_*f*_, the characteristic frequency follows *f*_*c*_ ∝ 1/*a*^2^ scaling almost exactly (Fig. 3(b) inset). This scaling is also verified separately by folded stainless steel mesh cubes of different sizes (Fig. S6). A more rigorous test of Eq. (2) ${f}_{c} \sim \frac{{\sigma }_{m}}{\pi {C}_{0}}\frac{3}{{a}^{2}{s}_{f}}$ involves two additional materials with different sizes (*a*) and significantly different surface areas: Regal 660 (*s*_*m*_ ∼ 90 *m*^*2*^*/g*)^45^ and Darco 12–20 (*s*_*m*_ ∼ 600 *m*^*2*^*/g*)^44^ as in Fig. 3(a). By plotting *f*_*c*_ against $\frac{3}{{a}^{2}{s}_{f}}$ in Fig. 3(b), we not only validated Eq. (2) quantitatively, but also measured *C*_0_ ∼ 3 *μF*/*cm*^2^ for porous carbon that is consistent with literature values^47,48^.

In addition to the change in *f*_*c*_, porous conductors also distinguish themselves by an asymmetric spectral peak (Fig. 3(a)), and this remarkable difference is fully consistent with our theoretical prediction as demonstrated in Fig. 3(a)-inset contrasting the response between porous conductors and solid conductors. In particular at high frequencies, unlike solid conductors with *ϕ* ∝ 1/*f*, porous conductors have a charge-discharge cycle that can only penetrate the grains up to a diffusive skin depth $\delta  \sim a\sqrt{{f}_{c}/f}$, which results in a much slower spectral decay: $\phi \propto 1/\sqrt{f}$. It is also worthwhile to note that the unique and distinguishable spectral IP response of these low-cost, off-the-shelf porous conductors make them a worthwhile candidate to evaluate for electromagnetically detectable tracer material use in drilling and completions applications^49^.

### Nano-porous overgrowths of framboidal pyrite linked to hydrocarbon occurrence

**Figure 4:**
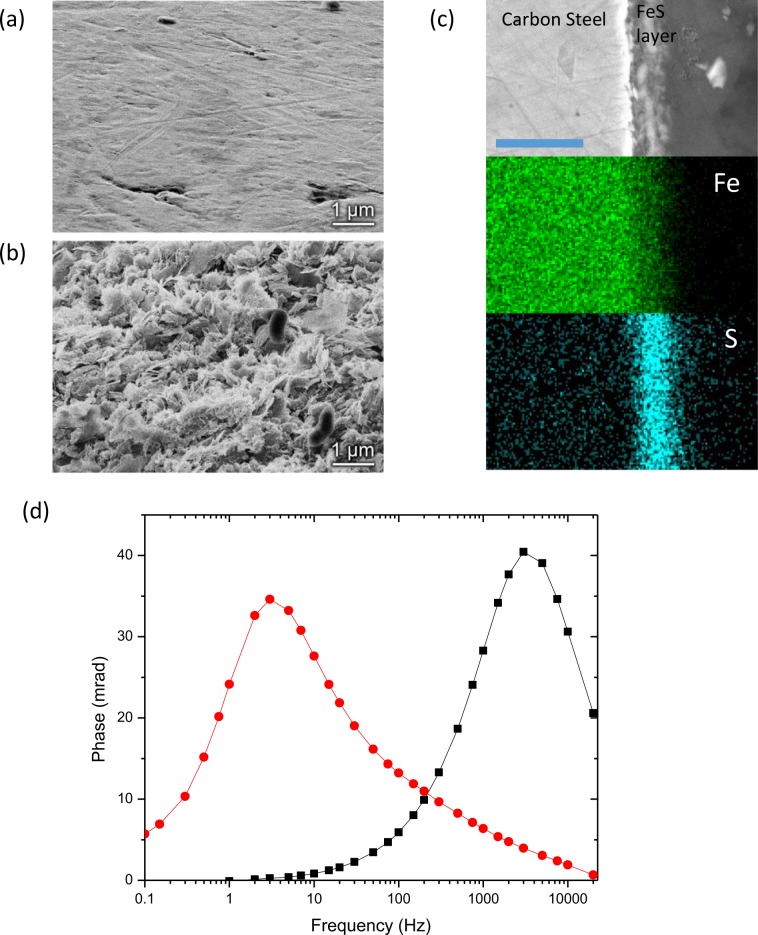
Induced Polarization of Nano-porous FeS from bacterially induced corrosion. (**a**,**b**) Helium ion microscopy images of untreated 1.6 mm carbon steel beads (**a**) and nano-porous FeS layer after bacteria-induced corrosion (**b**). Scale bars are 1 μm. (**c**) SEM and EDS cross section images of a carbon steel bead after formation of the FeS layer. Scale bar is 10 *μ*m. (**d**) Induced polarization spectrum of bare steel beads (black squares) from (**a**) and beads covered with nano-porous FeS layer (red circles) as in (**b**). The remarkable characteristic frequency reduction results from the porous conductive layer with pore scale ~$25$ to $50\,{\rm{nm}}$, qualitatively consistent with the observation in (**b**).

Our improved fundamental understanding of the electrode polarization mechanism and the role of nanoporosity can now be applied to petroleum basins in a marine setting, where a specific type of porous conductive material, nano-porous pyrite, can be biologically formed in saline subsea environments by anaerobic sulfate reducing bacteria^42^. One specific example is the conventional framboidal pyrite (typically has an overall size of 5–100 μm, and contains large quantities of sub-micron pyrite crystals) with a nano-porous pyrite overgrowth due to the activity of sulfate reducing bacteria in the presence of hydrocarbons^43,50^. Understanding the IP response from these porous conductive materials could potentially lead to new methods to find hydrocarbon seeps or migration pathways. To illustrate the origin and electrical response of this nano-porous layer, we performed a biomimetic experiment with 1.6 mm carbon steel beads in the presence of sulfate reducing bacteria in an anaerobic environment (see Methods). Growing on the nominally flat surface of bare carbon steel beads (Fig. 4(a)), the bacteria anaerobically respired sulfate, reducing it to H_2_S^51^ that led to the formation of a nano-porous FeS layer as a corrosion product. Helium ion microscopy (HIM) of the surfaces (Fig. 4(b)) revealed an irregularly-packed flaky morphology with sub-100 nm features (see higher-resolution figure in SI-Figure-S9). The thickness and composition of this nano-porous layer was measured in cross section with scanning electron microscopy (SEM) and energy-dispersive x-ray spectroscopy (EDS) (Fig. 4(c)) and was confirmed to be FeS at the surface with x-ray photoelectron spectroscopy (XPS). The frequency domain IP spectra shows a remarkable shift in frequency peak in Fig. 4(d), from *f*_*c*_ ∼ 3500 Hz that is expected for 1.6 mm carbon steel beads, to *f*_*c*_ ∼3 Hz for steel beads with a 5 *μ*m-thick nano-porous conductive layer, consistent with our theory based on the observed nanoporous morphology (See Methods).

In subsea sediments, it is possible to detect IP features of different forms of pyrite^14^, but in the absence of massive ore deposits associated with significant hydrothermal or volcanic activities^52–54^, we think that nano-porous pyrite is the only form of pyrite that would both correlate with hydrocarbon presence and would give rise to a distinguishable signature with the long relaxation times required for detection in conventional IP surveys. To form such specific nano-porous conductive structures, it is necessary to have sulfate reducing bacteria that produce H_2_S with the consumption of sulfate (${{{\rm{SO}}}_{4}}^{2-}$) and hydrocarbon (C_x_H_y_). The *in-situ* H_2_S production from anaerobic bacterial activity then reacts with different iron species and eventually forms nano-porous pyrite. In anoxic water columns, sulfate species diffuse downward from the seafloor, and are consumed by anaerobic bacteria in the sulfate reduction zone^42^. This process can also occur in euxinic water columns. In addition, mobile and constant hydrocarbon feed is necessary to form large clusters of framboidal pyrites with nano-porous overgrowths^15,43^ and aggregates^42^, as the conversion from localized organic carbon to pyrite is in some ways limited^55^ and the resulting euhedral and framboidal pyrites are relatively small.

The biomimetic experiment (Fig. 4) along with the fundamental physics discovered here provide the heretofore missing structure-property relationships that are necessary to infer the correct IP relaxation time scales of nano-porous aggregates, and also provide a potential mechanism to explain the long relaxation time^7^ observed in the geological setting with sedimentary pyrite formation above a hydrocarbon reservoir. For large solid euhedral pyrite(*a* ∼ 1 cm) or conventional framboidal pyrite (< *r*_*p*_ > ∼ 100 nm, *a* ∼ 10 *μ*m) without any nano-porous pyrite overgrowth, we can use Eqs. (1) or (2), respectively, to calculate a relaxation time *τ* < 10 ms under representative conditions (*σ*_*m*_ ∼ 0.2 S/m and *C*_*0*_ ∼ 30 *μ*F/cm^2^). Similar to the earlier results with euhedral pyrite in Fig. 1(b), our initial testing with conventional framboidal pyrite aggregates isolated from organic-rich sapropel layers with some levels of biological activities indicate peak frequencies in the kHz range, consistent with the mechanism and morphology described here. (These samples of sapropel layers from the Mediterranean were acquired from British Ocean Sediment Core Research Facility (BOSCORF); See SI-Text-S4 and Fig. S7 for more detail). In contrast, under the same conditions, pyrite with nano-porous morphologies demonstrated through our biomimetic experiments (Fig. 4) and natural samples^43^ (< *r*_*p* _> ∼ 10 nm, *a* ∼ 10–200 *μ*m^15,42,43^) would experience IP relaxation times *τ* ranging from ∼10 ms to ∼2 s assuming that their metallic nano-porous structures are inter-connected and filled with electrolytic conductive materials such as brine or clay. This mechanism based on nano-porous pyrite provides a possible explanation of the long IP relaxation time^7^ about 0.5–5 seconds observed in geological settings in the absence of massive ore deposits. It remains to be seen whether natural subsurface grains with the nanoporosity required to obtain large relaxation times form ubiquitously within hydrocarbon seeps, which would potentially allow IP surveys to help identify hydrocarbon charge, seep, or migration pathways^7^ by locating these nano-porous pyrite aggregates.

### Implications for geophysical measurements

While most of our discussions so far are primarily focused on offshore marine environments, our understanding and interpretation of induced polarization can be readily applied to many other geophysical applications utilizing both frequency-domain and time-domain IP methods (See SI-Text-S3 for our time-domain analysis). Based on our theory (Eqs. 1 and 2 and SI-Text) and experimental validations, in order to make meaningful interpretations from field as well as borehole induced polarization data across various geophysical applications, additional information are generally required such as mineralogy, morphology and size distribution of the conductive particles as well as the brine conductivity. Here we provide general perspectives on utilizing IP surveys to acquire additional subsurface and/or materials information with key examples of geophysical applications.

In offshore marine settings, either with a basin-scale controlled-source electromagnetic survey^2,7^ or an ocean-bottom shallow inductive method^56,57^, it is possible to acquire induced polarization or complex conductivity information^7^. In these scenarios, the real part of the subsurface conductivity can be acquired through an inversion algorithm with proper constraints^58^, which provides a map of the effective conductivity *σ*_*m*_ of the porous media (without conductive grains) in the subsurface. In such environments, salinity is typically high with *σ*_*w*_ > 1*S*/*m*, and as a result “membrane polarization” is less relevant leaving “electrode polarization” as the main signal for interpretation. For example, if the size distributions of the pyrite or other mineral deposits could be approximated from basin analysis, even just within an order of magnitude, it is possible to use Eq. (2) with the measured characteristic frequency and relaxation time scale to generate a subsurface map of surface-area-to-volume-ratio ${s}_{f} \sim \frac{{\sigma }_{m}}{\pi {C}_{0}}\frac{3}{{a}^{2}{f}_{c}}$, thus providing key insights on the morphology of the pyrite grains or other mineral deposits in the depositional enviroments. Such information can then be used in conjunction with basin analysis and geo-bio-chemistry analysis to link to micro-biological activities and potential indirect connections to hydrocarbon seeps, potentially assisted with structural constraints from seismic stratigraphy and joint seismic-CSEM inversion.

In an electromagnetic-based land survey^3,25–27^ involving near surface fresh water, both “membrane polarization”^4,9,27^ from clay, sand, and other ion-selective materials and “electrode polarization” from conductive inclusions play a role in this low salinity environment. As a result meaningful interpretations of induced polarization signals would typically require additional information such as clay content distribution so that IP response from “electrode polarization” can be decoupled from “membrane polarization” with a correct mechanistic model. However, in near surface geological settings with a known type of massive mineral/ore deposit^52–54^, “electrode polarization” could potentially be the primary source of the measured IP signals. This electrode polarization response can easily be quantified when the mineral grain size distribution is relatively narrow. In this case the grain size can be estimated from $a=\frac{{\sigma }_{m}}{\pi {f}_{c}{C}_{0}}$ with an inverted *σ*_*m*_ and mineral information (*C*_0_). The abundance or volume fractions of the conducting grains can also be directly tied to the maximum phase shift of IP response as in ${\phi }_{c}=\frac{9}{4}\frac{{V}_{cond}}{1+3{V}_{cond}}$ regardless of the brine conductivity. If the size distribution is wide, the convoluted spectral IP response can also be used to estimate the size range and abundance of conductive grains based on equation-S2 in the SI. In principle, the above mentioned methods can also be used in offshore marine settings with the appropriate understanding of geological settings and sedimentary structures.

Like all electromagnetic-based geophysical surveys, the electromagnetic and IP signal strength will be limited by energy dissipation in conductive media, as electromagnetic waves in subsurface attenuate exponentially with the travelling distance. This attenuation length or skin depth is of the form of $\delta =\sqrt{\frac{1}{\pi \,f\,\mu {\sigma }_{m}}}$ (*σ*_*m*_ is the effective conductivity of the porous media and *μ* is the magnetic permeability). Using this attenuation length we provide some perspectives to outline the detectability limitations of some specific materials (e.g. euhedral, framboidal and nanoporous pyrites) in different types of geophysical applications (e.g. offshore, on-land and borehole)

For conventional framboidal pyrite typically existing in subsea organic-rich layers (SI-Text-S4) or oil/gas reservoirs^42^, the detectability from a basin-scale or borehole IP survey depends highly on their sedimentary environment and in particular the salinity. We have demonstrated that the characteristic frequency of their IP response is about 5000 Hz (SI-Text-S4) in high salinity (3 wt% NaCl) translating to a skin depth of about 7 meters. The small skin depth in high salinity environments makes such conventional framboidal pyrites difficult to detect with a basin scale offshore or land survey (km-scale or larger). In this scenario, proper IP or complex conductivity interpretation in borehole logging^59^ can potentially take advantage of such a frequency range and skin depth and could be particularly useful in identifying the content and morphology of pyrite and other minerals in organic layers or oil/gas reservoirs. To contrast, in low salinity or freshwater^58^ (similar to 0.03 wt% NaCl), the characteristic frequency of the same pyrites would be about 50 Hz leading to a skin depth of 700 meters due to both the frequency and conductivity changes. This large skin depth in the shallow freshwater condition could enable the acquisition and interpretation of meaningful IP response for identifying the abundance and potentially morphology of the shallow mineral species, assuming the minerals such as pyrite are relatively abundant in a local region.

Unlike the conventional framboidal pyrites, pyrites with nanoporous morphologies are unique in the sense that even in marine environments with high salinity, these electronically conductive nanoporous structures can produce a uniquely low IP characteristic frequency (*f*_*c*_ ∼ 1 *Hz*) resulting in a large skin depth over 1000 meters under representative conditions such as *σ*_*m*_ ∼ 0.2 S/m for brine saturated porous media. In addition, to our knowledge, the nanoporous layers demonstrated through natural samples^43^ and our biomimetic experiments (Fig. 4) link directly to the anaerobic bacteria activity. Fed by hydrocarbons, these anaerobic bacteria respire sulfate and reduce it to H_2_S^51^ leading to the formation of a nanoporous conductive layer made of FeS or pyrite (FeS_2_). Given sufficient abundance, in principle such pyrite with nanoporous morphologies can be detected through offshore marine surveys and land surveys. Furthermore in borehole logging, along with resistivity, density, neutron porosity and other data, induced polarization data can potentially shed light on the nanoporous pyrite content and morphology and differentiate them from conventional framboidal pyrite from the unique frequency response.

## Conclusion

This study allows us to progress the interpretation of induced polarization measurements from the empirical and qualitative to the mechanistic and quantitative. We are now able to relate the measured spectral IP response to the abundance (volume fraction), morphology (size, and surface-area-to-volume-ratio) and intrinsic material properties (specific capacitance) of the conducting grains. The broad agreements between theory and experiments, and the applicability to time-domain analysis (See SI-Text-S3), suggest that our understanding can be readily applied to field-scale electromagnetic surveys for advanced IP mapping and interpretation. In geological settings, basin analysis can provide reliable insights into parameters such as porosity and brine concentration, and further analysis based on our IP method can be used to directly predict and map out useful mineralogical information, e.g., characteristic size and volume fraction of solid conductive inclusions. The formulation of the IP response from first principles presented here will enable development of structure-property relationships that can be used in partial-differential-equation based forward models and inversions, without resorting to empirical formulations that have made interpretation of induced polarization parameters difficult and subjective^1^. Furthermore, our discovery on the scaling of porous conductor suggests that a long relaxation time (~1 s) is a unique feature in sedimentary rocks containing nano-porous sulfides such as pyrite, which strongly correlates with the activity of sulfate reducing bacteria and hydrocarbon occurrence^6,7,14,15,43^. In addition to the broad geophysical applications, our discovery also leads to new strategies for surface area characterization of porous materials, super-capacitor design from biomimetic processes, and bio-inspired materials^47^.

## Methods

### Custom-designed four-probe measurement cell

As shown in Figs. 1(a) and S2, the gap between the bead pack with pyrite inclusions and the current electrodes is approximately 5 cm. The probe electrodes are made of gold coated copper with different geometries as in Fig. 1(a): the two current electrodes are square meshes with spacing about 3 mm to guarantee the uniformity of an electric field in the bead pack while preventing the accumulation of small bubbles; the two voltage electrodes are ring-shaped and only touching the cylindrical cell on the rim, with minimal metal in the current path.

### Electronics

National Instruments cards PXI-4461 and PXI-4462 (rates up to 200k Samples/s) are used respectively for signal generation and data acquisition along with National Instruments chassis (PXIe-1078). Two current electrodes of the measurement cell are connected to two symmetric 1000 *Ω* resistors that balance the circuits. We obtain the injected current by measuring the voltages across the 1000 *Ω* resistors, and all the voltages are measured differentially through SR560 voltage pre-amplifiers with 100 *MΩ*/25 *pF* input impedance to minimize the capacitance coupling signals.

### Materials

Glass beads are purchased through Sigma-Aldrich; Cubic pyrites are purchased through Ward’s science; 304 and 316 stainless steel spheres are purchased through McMaster Carr. Sodium Chloride is purchased through Sigma-Aldrich.

### Conductor coatings

50 nm of Gold and platinum coating on various sizes of spheres are achieved by plasma sputtering deposition (Denton sputter coater Hummer X) with gold and platinum targets (99.99% pure from Anatech USA). The uniformity of coating on spherical objects is achieved by a wireless controlled shaking device inside the vacuum chamber during the coating process. Our theoretical derivation suggests that the polarization effect of a hollow metal sphere would be the same as the solid spherical metal, since the solid shell behaves as a Faraday cage and the polarization charge is present only at the outer metal surface.

### Surface-area-to-volume ratios for porous conductors with specific geometries

For solid spheres, *s*_*f*_  = 3/*a* and from Equation-2 we recover the earlier result with solid a conductive sphere.

For isotropic-shaped porous objects (radius *a*) composed of electronically-connected small conductive spheres (radius *r*_*p*_) with a porosity of *φ*, ${s}_{f}=\frac{3(1-\varphi )}{{r}_{p}}$. This consideration is also consistent with an increased surface capacitance ${C}_{s} \sim \frac{(1-\varphi ){a}^{3}}{{r}_{p}}{C}_{0}$ for a porous conductor as compared to *C*_*s*_ ∼ *a*^*2*^*C*_*0*_ for a solid metal sphere, and therefore leads to a peak frequency ${f}_{c} \sim \frac{{\sigma }_{m}}{\pi {C}_{0}}\frac{{r}_{p}}{(1-\varphi ){a}^{2}}$ for porous conductors.

For a thin nano-porous conductive layer (average pore size < *r*_*p*_ >, layer thickness *h*) on a solid metal bead (radius *a*), simple geometrical considerations suggest that the surface-area-to-volume ratio should be ${s}_{f} \sim \frac{9(1-\varphi )h}{ < {r}_{p} > a}$ which leads to a peak frequency of ${f}_{c} \sim \frac{{\sigma }_{m}}{\pi a{C}_{0}}\frac{ < {r}_{p} > }{3(1-\varphi )h}$.

### Nanoporous FeS scale from sulfate reduced bacteria coating

The wild-type Desulfovibrio vulgaris (D. vulgaris) was obtained from Judy Wall at the University of Missouri. Freezer stocks of D. vulgaris containing 10% glycerol were used to inoculate overnight cultures. The growth media contains 30 mM lactate, 30 mM sulfate, 8 mM MgCl_2_, 20 mM NH_4_Cl, 2.2 mM phosphate buffer, 0.6 mM CaCl_2_, 24 mM NaCO_3_, 0.02% resazurin, 0.06 mM FeCl_2_, trace elements and Thauer’s vitamins, with pH adjusted to 7.2. Media was bubbled with 15% CO_2_ balance N_2_ and sodium dithionite was added immediately before inoculation to a final concentration of 1.5 mM. Overnight cultures of D. vulgaris were subcultured into serum bottles containing 80 ml of media and low-carbon steel balls (McMaster-Carr) and incubated at 30 °C overnight. After incubation, one set of carbon steel balls were transferred to 3 wt% NaCl solution for induced polarization measurement, while we process the other set with critical point drying and then use it for helium ion microscopy imaging.

### Correlating IP results to nanoporous FeS scale

From our earlier theoretical calculation ${f}_{c} \sim \frac{{\sigma }_{m}}{\pi a{C}_{0}}$, independently measured parameters (*f*_*c*_ ∼ 3 Hz, *h* ∼ 5 μm, *a* = 0.8 mm, and *C*_*0*_ ∼ 30 μF/cm^2^ for pyrite), and by assuming a reasonable range of porosity (0.1 < *φ* < 0.5), we infer an average pore size < *r*_*p*_ > ∼ 25–50 *nm*, demonstrating the consistency between induced polarization measurements and the helium ion images in Fig. 4(b).

## Supplementary information

Supplementary Information.
