# Supplementary material for: Quantifying Induced Polarization of Conductive Inclusions in Porous Media and Implications for Geophysical Measurements
Source: Sci Rep. 2020 Feb 3;10:1669. doi: 10.1038/s41598-020-58390-z (PMC6997379; doi:10.1038/s41598-020-58390-z)
Supplement: Supplementary file 1 — Supplementary Information. [file 41598_2020_58390_MOESM1_ESM.pdf]

# Supplementary Information for

## Quantifying Induced Polarization of Conductive Inclusions in Porous Media and Implications for Geophysical Measurements

Lang Feng[*1], Qiuzi Li[1], Steve Cameron[1], Kuang He[1§], Robert Colby[1], Katie Walker[1], Harry Deckman[1], Deniz Ertaş [*1]

[1]Corporate Strategic Research, ExxonMobil Research and Engineering, 1545 Route 22 East, Annandale, NJ, 08801, USA

§ Current Address: Sofinnova Partners, 7-11 Boulevard Haussmann, 75009 Paris, France

Corresponding Authors:
*Lang Feng lang.feng@exxonmobil.com;
*Deniz Ertaş deniz.ertas@exxonmobil.com

**This document includes:**

**Supplementary Materials - Text S1 to S5**

S1. Complex Conductivities of Solid Conductive Inclusions in Brine

S2. Effective Medium Theory of Complex Conductivity for Porous Conductive Inclusions in Brine

S3. Connecting Time-Domain and Frequency-Domain Induced Polarization Response

S4. Induced Polarization of Sedimentary Samples Containing Pyrite Framboids

S5. Local Formation Factor Calculation

**Supplementary Methods**

**References for SI**

**Supplementary Figures -** Figs. S1 to S9

**Supplementary Table** – Table. S1

## Supplementary Materials Text

### S1. Complex Conductivities of Solid Conductive Inclusions in Brine

Here we calculate induced polarization response in the limit where the conductivity of the conductive inclusion is much larger than the conductivity of the surrounding brine, and in the absence of redox-active ions, so that the inclusion can be treated as an "ideal polarizable electrode (IPE)"(See pages 11-13 in reference[1]).

Consider a conductive inclusion in the form of a single sphere of radius R of infinite conductivity, centered at the origin, surrounded by a brine with conductivity $\sigma_0$. When no external field is present, the electric double layer (EDL) in the sphere-brine interface equilibrates with a spontaneous potential $\phi_{sp}$ across the EDL. Typically in regimes of interest to us $\phi_{sp} \gg kT/e$, so the diffuse layer is saturated and any subsequent change in the potential across the interface requires an excess ionic surface charge density $\rho_{ex}$ in the Stern layer, balanced by an electronic image charge density of opposite polarity, by an amount controlled by the differential capacitance $C_0$: (See pages 540-553 in reference[1])

$$\rho_{ex} = C_0\left(\phi_s - \phi_{el} + \phi_{sp}\right).$$

Here, $\phi_s$ and $\phi_{el}$ are the potentials just outside and inside the EDL, respectively. $C_0$ is a material property that depends on the mineralogy of the conductive surface, presence of any dielectric film on the surface, the stoichiometry of the ionic species and the specific types of ionic species in the aqueous solution. Typical values of $C_0$ are on the order of $\sim 10\ \mu\mathrm{F/cm^2}$.

The $f \propto \sigma_w \sim n$ dependence ($n$: molar salt concentration) shown in main text figure 1(c) is experimental evidence that for frequencies far below the Debye relaxation

frequency $\sigma_w/\varepsilon$, the surface capacitance is indeed controlled by polarization in the Stern layer, rather than the diffuse layer. If we consider the traditional scaling with various double layer differential capacitance models [2-4], the major contribution arises from diffuse layer capacitance $C_D \propto \frac{\varepsilon_r \varepsilon_0}{\lambda_D}$, with vacuum permittivity $\varepsilon_0$, relative permittivity of water $\varepsilon_r$, and Debye screening length $\lambda_D$. With a dominating resistance $R \propto \frac{1}{\sigma_w}$ and a first-order relation $\frac{\varepsilon_r \varepsilon_0}{\sigma_w} = \frac{\lambda_D^2}{D}$ in the electrolyte, one would expect that the characteristic frequency of our system should scale as: $f \sim \frac{1}{RC_D} \sim \frac{\sigma_w \lambda_D}{\varepsilon_r \varepsilon_0} \sim \frac{D}{\lambda_D} \propto n^{0.5}$ with small higher-order corrections ($n$: molar salt concentration). The discrepancy between an approximate $f \propto n$ dependence in the measured figure 1(c) and a scaling relation $f \propto n^{0.5}$ suggests that the Guoy-Chapman-Stern model (See page 553 in reference[1]) provides the correct description: differential capacitance at high salinities are associated with charge accumulations in the Stern layer. Further experiments with model systems reported here have validated the Stern layer capacitance mechanism and allowed us to determine representative values for $C_0$ for gold, silver, platinum, various grades of steel, pyrite, and porous carbon. This is in stark contrast to mechanistic models in the literature [2-5] that relate characteristic frequencies to effective grain sizes, $a$, and ionic diffusivities, $D$, without regard to the overall conductivity of the brine.

Let us now consider the case where external fields are present, but they vary slowly enough and give enough time for the EDL to locally equilibrate. If the sphere is electronically isolated and infinitely conducting, it will act as an internal short circuit and its potential will float with the surrounding brine's potential, such that along the surface, $\phi_{sp} = \phi_{el} - \overline{\phi}_s$ , and consequently,

$$\rho_{ex} = C_0(\phi_s - \overline{\phi}_s),$$

where the overbar denotes an average over the entire surface area of the sphere. $\overline{\phi}_s$ can be set to zero in the subsequent discussion and will be dropped henceforth.

In the brine, the ionic current is given by

$$\mathbf{j} = -\sigma_0 \nabla \phi(\mathbf{r}).$$

Local charge neutrality outside of the EDL requires that $\nabla \cdot \mathbf{j} = 0$, thus the electric potential outside the sphere satisfies Laplace's equation:

$$\nabla^2 \phi(\mathbf{r}) = 0, \;\; r > R.$$

The boundary conditions just outside the EDL can be obtained from local conservation of ionic charge:

$$\frac{d\rho_{ex}}{dt} = \mathbf{j} \cdot \mathbf{n},$$

where $\mathbf{n}$ is the unit normal vector on the surface of the sphere, pointing outward. Thus, in the thin EDL limit,

$$\frac{\partial \phi}{\partial t} = -\frac{\sigma_0}{C_0}\frac{\partial \phi}{\partial r}, \qquad r = R_+$$

In spherical coordinates, for a spatially uniform and harmonically applied external electric field $E_0 e^{-i\omega t}$ in the z-direction, the solution is given by:

$$\phi(r,\theta;\omega) = -\left(E_0 r + E_i \frac{R^3}{r^2}\right)\cos\theta\, e^{-i\omega t}, \quad r > R$$

Here, $\theta$ is the polar angle between the position vector and the applied electric field, and $\omega$ is the angular frequency of excitation. The effective induced field amplitude $E_i$ is obtained by substituting this solution to the boundary condition just outside the EDL:

$$i\omega(E_0 + E_i)R = \frac{\sigma_0}{C_0}(E_0 - 2E_i)$$

Defining $\omega_0 \equiv 2\sigma_0/RC_0$ (or $f_c \equiv \sigma_0/\pi RC_0$), we get:

$$E_i = \frac{\omega_0 - 2i\omega}{2(\omega_0 + i\omega)} E_0$$

Outside the sphere, the axial component of the ionic current density is given (in terms of the current density $j_0 \equiv \sigma_0 E_0 e^{-i\omega t}$ in the absence of the sphere) by:

$$j_z = -\sigma_0 \nabla\phi \cdot \hat{\boldsymbol{z}} = j_0\left(1 + \frac{E_i}{E_0}\frac{R^3}{r^3}(1 - 3\cos^2\theta)\right), \qquad r > R.$$

Inside the sphere, there is a uniform axial electronic current that carries the image charges across the sphere:

$$j_z = j_0\left(1 - \frac{2E_i}{E_0}\right), \qquad r < R$$

Thus, the relative change in the axial current density due to the presence of the sphere is:

$$\frac{\delta j_z}{j_0} = \begin{cases} -\dfrac{\omega_0 - 2i\omega}{\omega_0 + i\omega}, & r < R \\ \dfrac{\omega_0 - 2i\omega}{2(\omega_0 + i\omega)} \dfrac{R^3}{r^3} (1 - 3\cos^2\theta), & r > R \end{cases}$$

Now consider a dilute collection of isotropically distributed conductive spheres embedded in a matrix with ionic conductivity $\sigma_0$, with a total volume fraction $v_0 \ll 1$. Neglecting the interactions between individual spheres, the macroscopic conductivity can be obtained by volume-averaging the gradient of the potential and current density over the position of the spheres. The contributions from outside the sphere vanish after averaging over the angle. Integrating the Electric field and the current inside the sphere yields:

$$\frac{\langle \delta E_z \rangle}{E_0} = v_0 \frac{E_i}{E_0}$$

$$\frac{\langle \delta j_z \rangle}{j_0} = -v_0 \frac{2E_i}{E_0}$$

Thus, to linear order in the volume fraction,

$$\frac{\delta\sigma}{\sigma_0} = \frac{\langle \delta j_z \rangle}{j_0} - \frac{\langle \delta E_z \rangle}{E_0} = -v_0 \frac{3(\omega_0 - 2i\omega)}{2(\omega_0 + i\omega)}$$

And as a result the effective complex conductivity can be simplified as:

$$\sigma_{eff} = (\sigma_0 + \delta\sigma) = \sigma_0 \left[1 + 3v_0 (1 - \frac{3}{2} \frac{1}{1 + if/f_c})\right], \qquad \text{(S1)}$$

with characteristic frequency $f_c \equiv \sigma_0/\pi R C_0$. Note the in the main text, $\sigma_0, R, v_0$ are denoted as $\sigma_m, a, V_{cond}$. Thus, at low frequencies ($f \ll f_c$) the spheres effectively act as

insulators and the conductivity is reduced by a factor of $1 - 3v_0/2$, whereas at high frequencies the conductivity is enhanced by a factor $1 + 3v_0$. The phase angle $\varphi$ between the current and voltage exhibits a maximum at $f = f_c$, with a value $\varphi_{max} = \varphi(f_c) = 9v_0/4$.

For polydispersed spherical inclusions with a volume-weighted size distribution $p_V(R)$, the complex conductivity is given by:

$$\sigma_{eff} = \sigma_0 \left[1 + 3v_0 \int_0^\infty dR\, p_V(R) \left(1 - \frac{3}{2}\frac{1}{1 + if/f_c(R)}\right)\right]. \qquad \text{(S2)}$$

For such cases, fitting measurements to the Cole-Cole Model is likely to yield a result with an effective exponent $c < 1$.

At small volume fraction ($v_0 \ll 1$), the key physical scaling can be understood intuitively in light of the RC circuit model shown in figure S2(b) with connections to fundamental physical parameters with a simple electrolytic capacitor $C_s$ whose nominal capacitance is independent of frequency, i. e., a Cole-Cole response with exponent $c = 1$. Consider a slab of area $A \sim a^2/v_0$ and thickness $\sim a$ that is normal to the direction of the applied electric field. The slab includes on average one conducting grain, which acts as an electrolytic capacitor of capacitance $C_s \sim a^2 C_0$. The current flowing through this slab can be partitioned into a current that flows through the capacitor across a resistance $R_2 \sim \frac{1}{a\sigma_m}$ , and a current that does not flow through the conductor and instead encounters a background resistor $R_1 \sim \frac{1}{\sigma_m}\frac{a}{A} \sim v_0 R_2$ . Substituting these in the circuit model, we obtain $f_c' = \frac{1}{2\pi R_2 C_s} \sim \frac{\sigma_m}{aC_0}$ and $\phi_c' \sim \frac{R_1}{R_2} \sim v_0$. In general we expect the numerical prefactors to depend on the particular shape of the conducting grains, based on the full derivation described earlier in this section.

## S2. Effective Medium Theory of Complex Conductivity for Porous Conductive Inclusions in Brine

Framboids are spheroidal agglomerations of much smaller primary particles. Internally, they can be thought of as a porous medium, characterized by an interparticle porosity $\phi_f$ and a surface to volume ratio $s_f \sim \alpha_p/r_p$ related to the characteristic size $r_p$ and relative surface roughness $\alpha_p$ of the primary particles. Brine filling the pores of the framboid is assumed to be the same brine that surrounds the framboid, resulting in an effective ionic conductivity of $\sigma_f = \sigma_0/F_f(\phi_f)$, where $F_f$ is the formation factor of the framboid with a generalized Archie's Law. A fundamental assumption we will make about pyrite and other conducting framboids is that the primary particles are in electronic contact with each other, with an effective bulk electronic conductivity $\sigma_e$. Let us replace our sphere with a framboid of radius $R$ and revisit the problem solved in the previous section.

The outer solution will have the exact same form:

$$\phi_o(r,\theta;\omega) = -\left(E_0 r + E_i \frac{R^3}{r^2}\right)\cos\theta\, e^{i\omega t}, \quad r > R$$

However, for $r < R$ we now have an ionic conductor phase inside the framboid and need to solve the inner ionic potential $\phi_i$, as well as the electronic potential $\phi_{sp} + \phi_e$. The charging condition for the EDL dictates that the ionic and electronic currents satisfy:

$$\nabla \cdot \mathbf{j_i} = -\sigma_f \nabla^2 \phi_i = -C_0\, s_f \frac{\partial(\phi_i - \phi_e)}{\partial t}$$

$$\nabla \cdot \mathbf{j_e} = -\sigma_e \nabla^2 \phi_e = -C_0\, s_f \frac{\partial(\phi_e - \phi_i)}{\partial t}$$

Algebraic manipulation yields the following (with $\phi_{EDL} \equiv \phi_i - \phi_e$):

$$\phi_i = \frac{\sigma_e}{\sigma_e + \sigma_f} \phi_{EDL} + \phi_d,$$

$$\phi_e = -\frac{\sigma_f}{\sigma_e + \sigma_f} \phi_{EDL} + \phi_d,$$

where the potential $\phi_{EDL}$ across the EDL satisfies the diffusion equation, and the drift potential $\phi_d$ satisfies the Laplace equation:

$$\frac{\partial \phi_{EDL}}{\partial t} = \frac{\sigma_f \sigma_e}{C_0 s_f \left(\sigma_f + \sigma_e\right)} \nabla^2 \phi_{EDL},$$

$$\nabla^2 \phi_d = 0.$$

At the boundary of the framboid, the boundary conditions are:

(i) Continuity of ionic potential: $\phi_i(R, \theta; \omega) = \phi_o(R, \theta; \omega)$:

$$\phi_i(R, \theta; \omega) = -(E_0 + E_i) R \cos\theta\, e^{i\omega t}$$

(ii) Continuity of ionic current normal to the boundary: $\sigma_f \left.\frac{\partial \phi_i}{\partial r}\right|_{r=R} = \sigma_0 \left.\frac{\partial \phi_o}{\partial r}\right|_{r=R}$:

$$\left.\frac{\partial \phi_i}{\partial r}\right|_{r=R} = -F_f (E_0 - 2E_i) R \cos\theta\, e^{i\omega t}$$

(iii) Continuity of electronic current normal to the boundary:

$$\left.\frac{\partial \phi_e}{\partial r}\right|_{r=R} = 0.$$

The solution is of the form

$$\phi_d(r,\theta;\omega) = -E_d r \cos\theta \, e^{i\omega t}$$

$$\phi_{EDL}(r,\theta;\omega) = -\frac{\sigma_e + \sigma_f}{\sigma_e}(E_0 + E_i - E_d)\frac{u(r/R)}{r/R} R\cos\theta \, e^{i\omega t}, \qquad r < R$$

where $u(\rho)$ satisfies (with $\rho \equiv r/R$, $\tilde{\omega} \equiv \omega/\omega_f$, where $\omega_f \equiv \frac{\sigma_f \sigma_e}{C_0 s_f (\sigma_f + \sigma_e) R^2}$)

$$u'' - \frac{2u}{\rho^2} - i\tilde{\omega} u = 0.$$

This linear second order homogeneous ODE has two independent solutions:

$$u = A\left(\frac{\cos\left(\rho\sqrt{-i\tilde{\omega}}\right)}{\rho\sqrt{-i\tilde{\omega}}} + \sin\left(\rho\sqrt{-i\tilde{\omega}}\right)\right) + B\left(\cos\left(\rho\sqrt{-i\tilde{\omega}}\right) - \frac{\sin\left(\rho\sqrt{-i\tilde{\omega}}\right)}{\rho\sqrt{-i\tilde{\omega}}}\right)$$

The coefficients $A$ and $B$ are obtained from the symmetry condition $\phi_i(r=0) = 0$, which implies $A = 0$, and the ionic potential continuity (i) which implies that $u(1) = 1$. The final solution is:

$$u(\rho) = \frac{\cos\left(\rho\sqrt{-i\tilde{\omega}}\right) - \left(\rho\sqrt{-i\tilde{\omega}}\right)^{-1}\sin\left(\rho\sqrt{-i\tilde{\omega}}\right)}{\cos\left(\sqrt{-i\tilde{\omega}}\right) - \left(\sqrt{-i\tilde{\omega}}\right)^{-1}\sin\left(\sqrt{-i\tilde{\omega}}\right)} \qquad (S3)$$

The effective induced field $E_i$ and the drift field $E_d$ are then obtained by continuity of ionic and electronic currents (ii) and (iii):

$$F_f(E_0 - 2E_i) = (E_0 + E_i - E_d)(u'(1) - u(1)\,) + E_d,$$

$$0 = -\frac{\sigma_f}{\sigma_e}(E_0 + E_i - E_d)(u'(1) - u(1)\,) + E_d.$$

Noting that $u'(1) - u(1) = \alpha^{-1}(\tilde{\omega}) - 2$ with $\alpha(\tilde{\omega}) \equiv \frac{\sqrt{-i\tilde{\omega}}\cos(\sqrt{-i\tilde{\omega}}) - \sin(\sqrt{-i\tilde{\omega}})}{i\tilde{\omega}\sin(\sqrt{-i\tilde{\omega}})}$ we get:

$$\begin{bmatrix} 2(F_f - 1) + \alpha^{-1}(\tilde{\omega}) & 3 - \alpha^{-1}(\tilde{\omega}) \\ -1 & \dfrac{F_f\alpha(\tilde{\omega})}{1 - 2\alpha(\tilde{\omega})}\dfrac{\sigma_e}{\sigma_0} + 1 \end{bmatrix} \begin{bmatrix} E_i \\ E_d \end{bmatrix} = \begin{bmatrix} F_f + 2 - \alpha^{-1}(\tilde{\omega}) \\ 1 \end{bmatrix} E_0$$

The general solution can be obtained and plotted for any desired value of parameters. Our main interest is once again in the limit where the conductivity ratio $\epsilon \equiv \frac{\sigma_0}{\sigma_e} \ll 1$. In this limit, $E_d \ll E_i$ and the equations can be simplified to yield:

$$\frac{E_i}{E_0} = \frac{\alpha(\tilde{\omega})(F_f + 2) - 1}{1 + 2\alpha(\tilde{\omega})(F_f - 1)}\big(1 + O(\epsilon)\big)$$

$$\frac{E_d}{E_0} = \epsilon\frac{3\big(1 - 2\alpha(\tilde{\omega})\big)}{1 + 2\alpha(\tilde{\omega})(F_f - 1)}\big(1 + O(\epsilon)\big)$$

Checking the low and high frequency limits where $\alpha$ approaches 1/3 and 0 respectively,

$$\frac{E_i}{E_0} = \begin{cases} \dfrac{F_f - 1}{2F_f + 1} & \tilde{\omega} \ll 1 \\ -1, & \tilde{\omega} \gg 1 \end{cases}$$

recovering the correct behavior at both limits.

Let us once again consider a dilute collection of uniformly distributed framboids with volume fraction $v_0 \ll 1$. Neglecting the interactions between individual framboids, the macroscopic conductivity can be obtained by volume-averaging the electric field and current density over the position of the spheres. The contributions from outside the sphere once again vanish after averaging over the angle. Integrating the Electric field and the currents inside the sphere yields (note that the EDL charging currents cancel out and only the drift current contributes to the volume integral):

$$\frac{\langle \delta E_z \rangle}{E_0} = v_0 \frac{E_i}{E_0} = v_0 \frac{\alpha(\widetilde{\omega})\left(F_f + 2\right) - 1}{1 + 2\alpha(\widetilde{\omega})\left(F_f - 1\right)} \left(1 + O(\epsilon)\right)$$

$$\frac{\langle \delta j_z \rangle}{j_0} = v_0 \left( \left( \frac{1}{F_f} + \frac{1}{\epsilon} \right) \frac{E_d}{E_0} - 1 \right) = v_0 \frac{2 - 2\alpha(\widetilde{\omega})\left(F_f + 2\right)}{1 + 2\alpha(\widetilde{\omega})\left(F_f - 1\right)} \left(1 + O(\epsilon)\right)$$

Thus, to linear order in the volume fraction,

$$\frac{\delta\sigma}{\sigma_0} = \frac{\langle \delta j_z \rangle}{j_0} - \frac{\langle \delta E_z \rangle}{E_0} = v_0 \frac{3\left(1 - \alpha(\widetilde{\omega})\left(F_f + 2\right)\right)}{1 + 2\alpha(\widetilde{\omega})\left(F_f - 1\right)}. \qquad \text{(S4)}$$

Since $\alpha(0) = 1/3$ and $\alpha(\infty) = 0$, The limiting values for the change in conductivity are:

$$\frac{\delta\sigma}{\sigma_0} = \begin{cases} -\dfrac{3v_0\left(F_f - 1\right)}{2F_f + 1}, & \omega \ll \omega_f \\ 3v_0, & \omega \gg \omega_f \end{cases}$$

At low frequencies the framboid will behave as an insulator so that the conductivity change is zero when the formation factor is 1 and -$3v_0/2$ when the formation factor is

infinity (back to the solid sphere case). At high frequencies the framboid acts as a short circuit and we again recover the behavior of the solid sphere, with a conductivity increase of $3v_0$.

Since the corrections to the conductivity are linear in the volume fraction, for grains with a distribution of shapes, sizes, or formation factors, the effective conductivity can be computed using the superposition principle, as was done for the case of solid grains in the preceding section. For more complex geometries, the equations and boundary conditions provided here allow construction of  mechanistic, PDE-based forward models, rather than empirical Cole-Cole type Induced Polarization models used in the literature.

Let us now look at two examples:

Packing of spheres: If the framboid microstructure can be approximated by a packing of (smooth) spheres with uniform radius $r_p$ , porosity $\phi \approx$ 0.4 and Formation Factor $F_f \approx$ 3, then

$$s_f = \frac{3(1-\phi)}{r_p} \approx \frac{1.8}{r_p}$$

Compared to the characteristic frequency of the corresponding solid conductive sphere,

$$\omega_f = \frac{\sigma_0 r_p}{3(1-\phi)F_f C_0 R^2} \approx \omega_0 \left(\frac{r_p}{10.8\, R}\right)$$

Porous Stainless Steel Meshes: In another example, we tested porous cubes of folded stainless-steel meshes and porous carbon beads. The same stainless-steel meshes are cut and folded into two different sets of cubes, one large cube with edge length 1.5 to 2 cm

and four small cubes with 0.6 to 0.8 cm, and both sets have approximately the same folding density (20 plates/mm) to ensure a constant $s_f$. Based on our measurements in figure 2(c) and equation 1 in the main text, a solid 304 stainless steel cube of 1.5 to 2cm would have a frequency peak $f_c \sim 270Hz$ and $f_c \propto 1/a$, while in Figure S6 we show that the porous cube of the same size experienced a much lower peak frequency $f_c \sim 45Hz$ due to higher surface area. With small cubes (0.6 to 0.8 cm) that are ~2.5 times smaller in size ($a$), this peak frequency goes up almost 8 times to $f_c \sim 350Hz$ and clearly follows the new scaling $f_c \propto 1/a^2$.

We obtained further validation of the scaling form (S3)-(S4) using commercially available porous carbon beads of various sizes and specific surface areas (see main text, figure 3). Such particles could be used as tracer materials with distinct electromagnetic signatures for oilfield applications [6].

## S3. Connecting Time-Domain and Frequency-Domain Induced Polarization Response.

It is relatively uncommon to deploy frequency domain EM surveys to detect induced polarization response, due to challenges associated with accurate measurement of phase [7,8]. Therefore, time domain EM surveys are typically used to achieve "chargeability" and "relaxation time" maps to identify minerals [9-12]. Time-domain and frequency domain results can be related through a Fourier Transform, and for our particular RC circuit model, the chargeability $m$ is related to the maximum phase shift $\phi_c$ via $2\tan\phi_c = \frac{m}{\sqrt{1-m}}$, and the relaxation time is $\tau_{TD} = \frac{1}{2\pi\sqrt{1-m}f_c}$. In figure 3 we demonstrate this

connection by experimentally comparing time-domain and frequency-domain measurements with both low and high volume fractions. For time domain measurements, we use a Valhalla current source to generate a current square wave with a rise time $\sim 100\mu s$, and monitor the voltage across the sample (see Supplementary Materials and Methods for more detail). An example of low volume fraction measurements is shown in figure S5(a) with 304 stainless steel (6.4mm) at 0.3 wt% NaCl. Based on the frequency domain results (figure S5(a) inset): $f_c = 52.7$ Hz and $\phi_c = 70.7$ mrad, which correspond in time domain to $\tau_{predict} = \frac{1}{2\pi f_c} = 3.0$ ms and $m_{predict} = 2\phi_c = 14.2\%$. As expected the measured voltage does not track the current trace but has an exponential tail due to induced polarization. The parameters that we extracted from the exponential tail: $\tau_{measure} = 2.8 \pm 0.2$ ms and $m_{measure} = 14\% \pm 1\%$ are in excellent agreement with the frequency-domain response.

In figure S5(b), we show a similar example but with large volume fraction of pyrite inclusions (~20%) with 3 wt% NaCl. The frequency domain results $f_c = 44.0$ Hz and $\phi_c = 0.326$ rad from the inset correspond to $\tau_{predict} = 5.04$ ms and $m_{predict} = 48.6\%$, consistent with experimental measurements $\tau_{measure} = 4.9 \pm 0.2$ ms and $m_{measure} = 49\% \pm 1\%$.

**S4. Induced Polarization of Sedimentary Sample Containing Pyrite Framboids**

Sedimentary samples were obtained from the British Ocean Sediment Core Research Facility (BOSCORF) at the National Oceanography Centre, Southampton. We have selected three specific samples for extensive characterizations: (a) depth 117 cm to 123 cm within section 1 of core LC32 from cruise MD81 [13]; (b) depth 25 cm to 35 cm within

section 6 of core LC21 from cruise MD81; (c) depth 6 cm to 12 cm within section 1 of core VC08 from cruise JC77 [14]. These specific locations of the cores are chosen as they contain significant organic-rich sapropel layers that are known to correlate with the presence of framboidal pyrites due to diagenetic pyritization just beneath the sapropel layer. [13,15] The X-Ray diffraction (XRD) data of sample (a) shows the presence of gypsum ($CaSO_4$), quartz, and calcite ($CaCO_3$), and minor pyrite, clays, and halite (NaCl). The XRD data of sample (b) shows the presence of quartz and calcite as the major phases, and minor pyrite, halite and clay minerals in the kaolinite, mica, and chlorite families. The XRD data of sample (c) shows the presence of mostly quartz, some pyrite and minor feldspar, calcite and clays. In all XRD data we do not see evidence of other iron minerals such as siderite ($FeCO_3$), magnetite ($Fe_3O_4$), hematite ($Fe_2O_3$), or goethite (FeO(OH)).

Induced polarization tests were conducted on either as-received samples, or samples in which pyrite was concentrated using a miniaturized induced polarization sample cell saturated with seawater (from Sigma-Aldrich). Pyrite concentrations were improved by using a sieving process (90 $\mu$m) in seawater to concentrate minerals and large aggregates, as in similar sedimentary enviroments the presence of pyrites is often associated with forams [13] that are often larger than 30 $\mu$m. We have also tried an alternative approach of concentrating materials containing pyrites by the use of a customized magnetic gradient concentrator. In this scenario, several cubic neodymium permanent magnets (1.2 cm cubes) were placed at the surface of a flow system, and the dispersed grains containing paramagnetic pyrites were attracted to the high magnetic gradient zones (corners of the cubic magnet), and then extracted and concentrated by careful rinsing and gravitational

separation. In either case, we have observed a large amount of forams, skeletal fragments, euhedral pyrites, and framboidal pyrites with Scanning Electron Microscopy (Figure S7(b)) and energy-dispersive X-ray spectroscopy. A representative induced polarization result with sample (a) after the sieving process is shown in figure S7(a) and figure S7(b). These figures show representative SEM images along with the predicted $f_c$ labeled in the images. The $f_c$ is calculated based on the effective medium theory in the previous section and also the assumption that pyrite microcrystals are electronically connected within a framboid. For samples (b) and (c) after either the sieving process or magnetic gradient concentration, induced polarization results show a small monotonic increment of phase shift at higher frequency, consistent with the fact that the predicted $f_c$ from representative SEM images are mostly above our frequency measurement range. The miniaturized sample cell has been tested against seawater and other conductive inclusions, and shows a noise floor of ~ 0.1 mrad in a frequency range from 5 Hz to 20 kHz.

**S5. Local Formation Factor Calculation**

In most scenarios, the formation factor $F$ in $\sigma_m = \frac{\sigma_w}{F}$ remains constant $F \sim 2.8 \pm 0.1$ for random packing of spheres if the volume fraction of metal is low and the metal size to glass bead size ratio $a/a_g \gg 1$. However when the size of metallic inclusions is comparable to that of the glass beads or cell diameter, the charge relaxation pathway around the metal deviates from that of global considerations, and in this case a local formation factor $F_L$ needs to be used to take into account the local geometry in the surrounding region ($\sim a$). Figure S8 shows measured characteristic frequency changes

with respect to $a/a_g$ for 1.6 mm diameter 316 Stainless Steel spheres in monodispersed glass bead packs with different sizes of glass spheres. Local corrections are not needed for data in figure 3 and S4 as in all gold and stainless steel experiments $a/a_g$ is kept above 5, however for small 0.15mm silver grains and the 17mm large pyrite cube, we estimate $F_L$ to be 1.4 and 6.7 respectively.

## Supplementary Methods

*Current Source: A Valhalla 2500 current calibrator is used in time domain tests to supply current square waves. The bandwidth of the calibrator is 10kHz, that leads to a rise time around* $\sim 100\mu s$ *in the generated current. In our control tests involving NaCl solutions and glass beads, the voltage and current square signals track each other within <0.1% difference, and show no induced polarization signature.*

## References for SI


1 Bard, A. J. & Faulkner, L. R. *Electrochemical methods: fundamentals and applications*. 2nd ed. edn, (Wiley, 2000).

2 Srinivasan, S. *Fuel cells: from fundamentals to applications*. (Springer Science & Business media, 2006).

3 Merriam, J. Induced polarization and surface electrochemistry. *Geophysics* **72**, F157-F166 (2007).

4 Lockett, V., Sedev, R., Ralston, J., Horne, M. & Rodopoulos, T. Differential capacitance of the electrical double layer in imidazolium-based ionic liquids: influence of potential, cation size, and temperature. *The Journal of Physical Chemistry C* **112**, 7486-7495 (2008).

5 Qi, Y. *et al.* Induced polarization response of porous media with metallic particles — Part 7: Detection and quantification of buried slag heaps. *GEOPHYSICS* **83**, E277-E291, doi:10.1190/geo2017-0760.1 (2018).

6 Wheelock, B., Ertas, M. D., Feng, L., Qiuzi, L. & Deckman, H. W. (US patent application US20180149020A1, 2018).

7 Sternberg, B. K. A review of some experience with the induced-polarization/resistivity method for hydrocarbon surveys: Successes and limitations. *Geophysics* **56**, 1522-1532 (1991).

8 Oehler, D. Z. & Sternberg, B. K. Seepage-Induced Anomalies. *AAPG Bulletin* **68**, 1121-1145 (1984).

9 Veeken, P., Kudryavceva, E., Putikov, O., Legeydo, P. & Ivanov, S. Modelling induced polarization effects due to pyrite in geochemical alteration zones above hydrocarbon accumulations. *Petroleum Geoscience* **18**, 59-72 (2012).

10 Flekkøy, E. G., Legeydo, P., Håland, E., Drivenes, G. & Kjerstad, J. in *The 2nd International CSEM Conference. CSEM in hydrocarbon exploration and exploitation, Oslo.*

11 Hodges, G. & Chen, T. Geobandwidth: comparing time domain electromagnetic waveforms with a wire loop model. *Exploration Geophysics* **46**, 58-63 (2014).

12 Yamashita*, Y., Lebert, F., Gourry, J.-C., Bourgeois, B. & Texier, B. in *SEG Technical Program Expanded Abstracts 2014* 1775-1779 (Society of Exploration Geophysicists, 2014).

13 Mercone, D. C. *High-resolution geochemical studies on the most recently-accumulated sapropel S1 in the eastern Mediterranean*, University of Southampton, (1999).

14 Lichtschlag, A., Cevatoglu, M., Connelly, D., James, R. & Bull, J. Increased fluid flow activity in shallow sediments at the 3 km Long Hugin Fracture in the central North Sea. *Geochemistry, Geophysics, Geosystems* **19**, 2-20 (2018).

15 Reinholdsson, M., Snowball, I., Zillén, L., Lenz, C. & Conley, D. Magnetic enhancement of Baltic Sea sapropels by greigite magnetofossils. *Earth and Planetary Science Letters* **366**, 137-150 (2013).

## Supplementary Figures

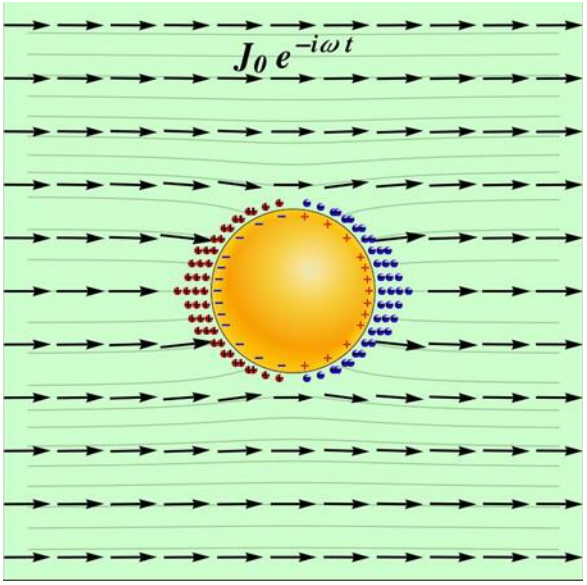


**Fig. S1.** Graphic illustration of induced polarization mechanism when a more conductive electronic conductor is surrounded by brine saturated porous media.

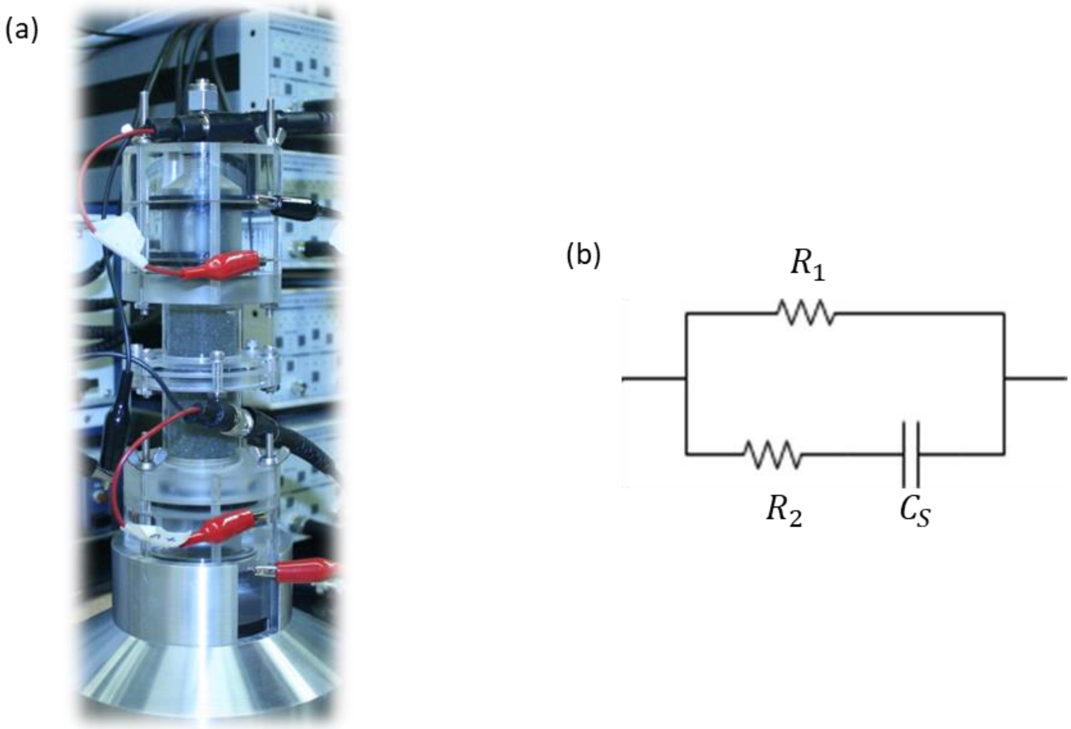


**Fig. S2**. (a) Four-probe experimental cell for electrical measurement of model porous media. (b) RC circuit for simplified physical scaling arguments.

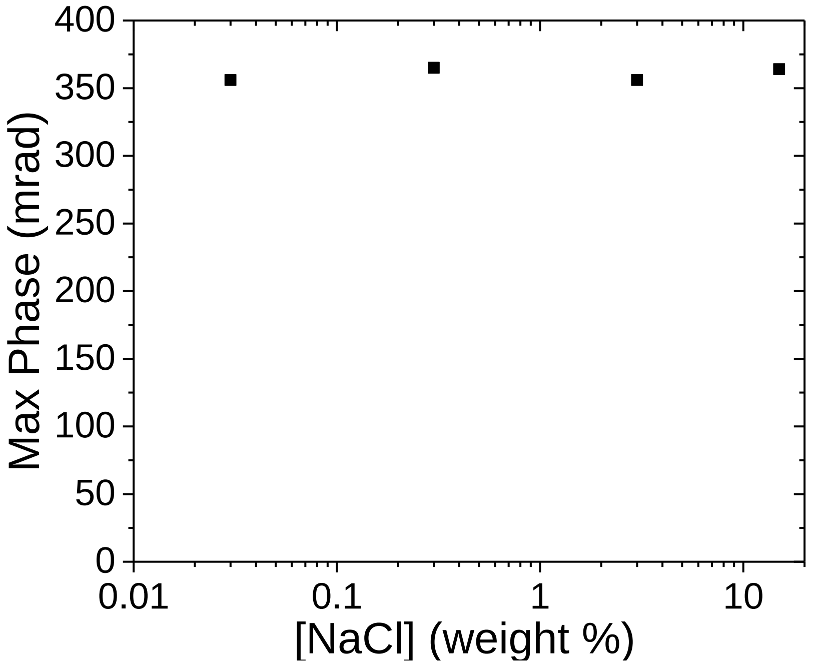


**Fig. S3.** Maximum phase shifts versus sodium chloride concentrations for the samples containing 2 pyrite cubes.

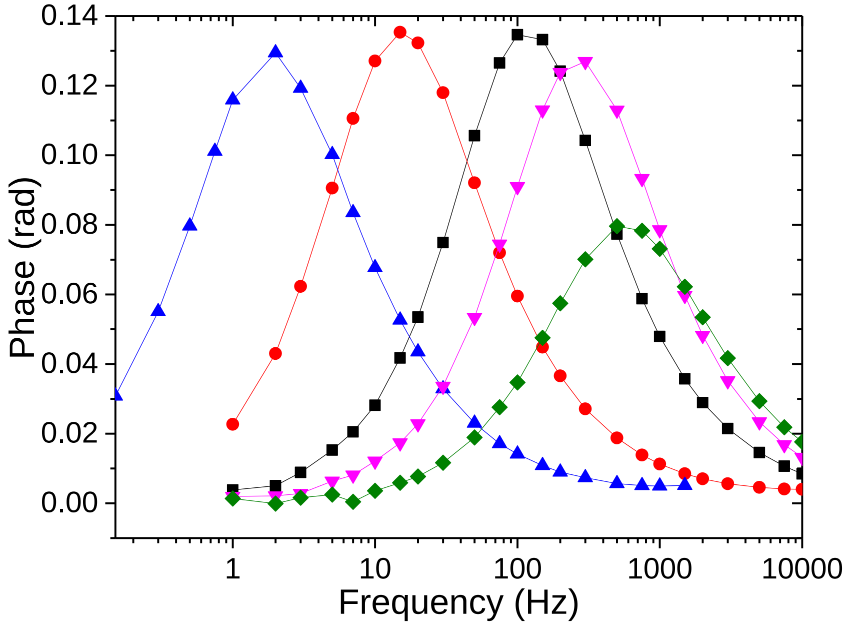


**Fig. S4.** Frequency dependent phase shift of glass bead pack with gold sphere inclusions: 7.6% v/v 4 mm gold spheres with 0.03 wt% NaCl (Blue triangles), 0.3 wt% NaCl (Red circles), and 3 wt% NaCl (Black squares); Pink inverse-triangles: 7.0% v/v 2 mm gold spheres with 3 wt% NaCl; Green diamonds: 4.9% v/v 4 mm gold spheres with 3 wt% NaCl.

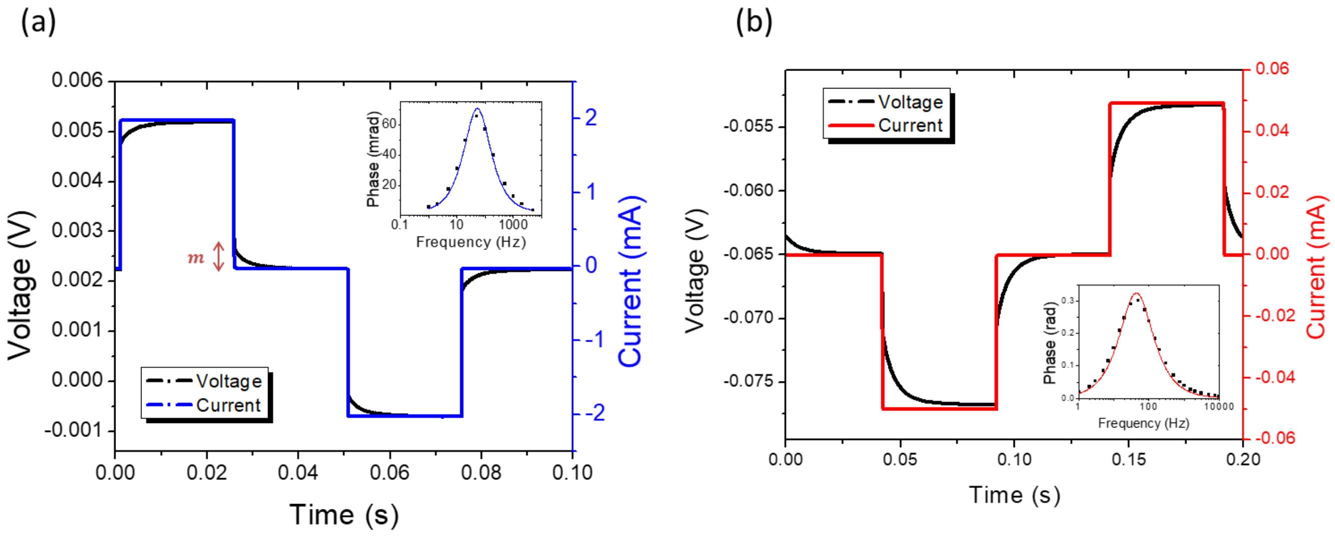


**Fig. S5. Time-Domain measurements and predictions**

(a) Time domain measurement with injected current square wave (blue) and probed voltage across the porous media (black), for 3.3% v/v 6.4 mm 304 stainless steel spheres with 0.3 wt% NaCl. The inset is the corresponding frequency-domain spectrum measurements with the same sample. The frequency domain results $f_c = 52.7$ Hz and $\phi_c = 70.7$ mrad from the inset correspond to $\tau_{predict} = \frac{1}{2\pi f_c} = 3.0$ ms and $m_{predict} = 2\phi_c = 14.2\%$. Time domain measurements of $\tau_{measure} = 2.8 \pm 0.2$ ms and $m_{measure} = 14\% \pm 1\%$ recover essentially the same result.

(b) Time domain measurement with injected current square wave (red) and probed voltage across the porous media (black lines), for ~20% v/v 17 mm cubic pyrite with 3 wt% NaCl. The inset is the corresponding frequency-domain spectrum measurement with the same sample. The frequency domain results $f_c = 44.0$ Hz and $\phi_c = 0.326$ rad from the inset correspond to $\tau_{predict} = 5.04$ ms and $m_{predict} = 48.6\%$, in excellent agreement with time-domain experimental measurements $\tau_{measure} = 4.9 \pm 0.2$ ms and $m_{measure} = 49\% \pm 1\%$.

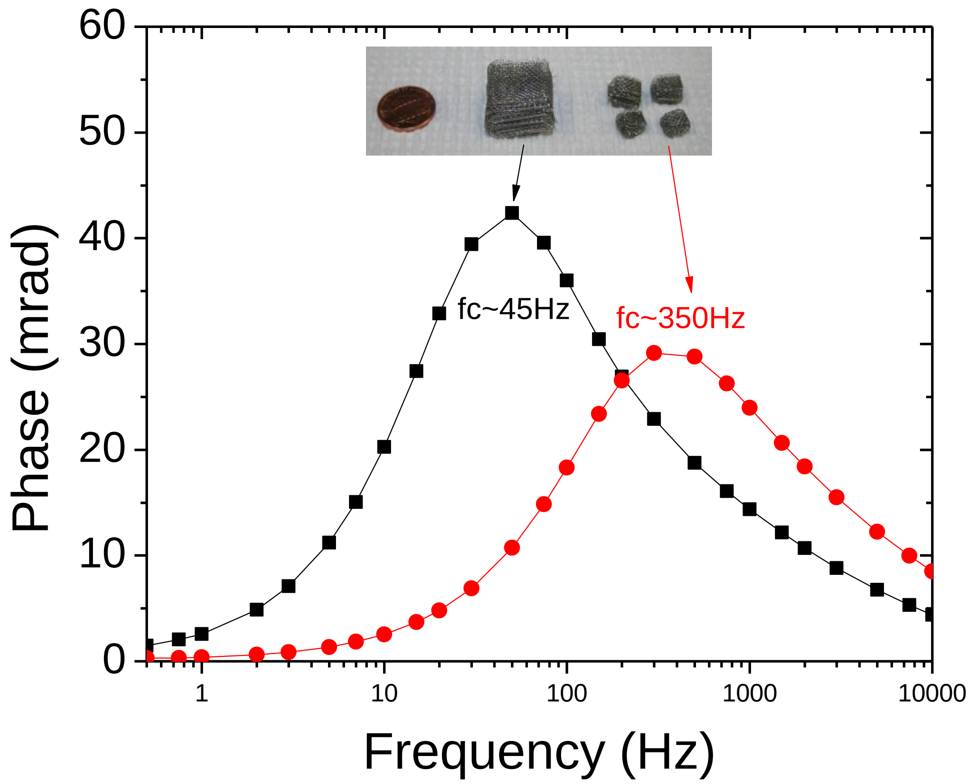


**Fig. S6.** A porous 304 stainless steel cube of 1.5 to 2 cm has a peak frequency $\boldsymbol{f_c} \sim \mathbf{45\ Hz}$ (black curve), while for small cubes (0.6 to 0.8 cm) that are ~2.5 times smaller in size (**a**), this peak frequency goes up almost 8 times to $\boldsymbol{f_c} \sim \mathbf{350\ Hz}$ (red curve) and clearly follows the new scaling $\boldsymbol{f_c} \propto \mathbf{1/\boldsymbol{a}^2}$. The penny on the left is a guide for scale.

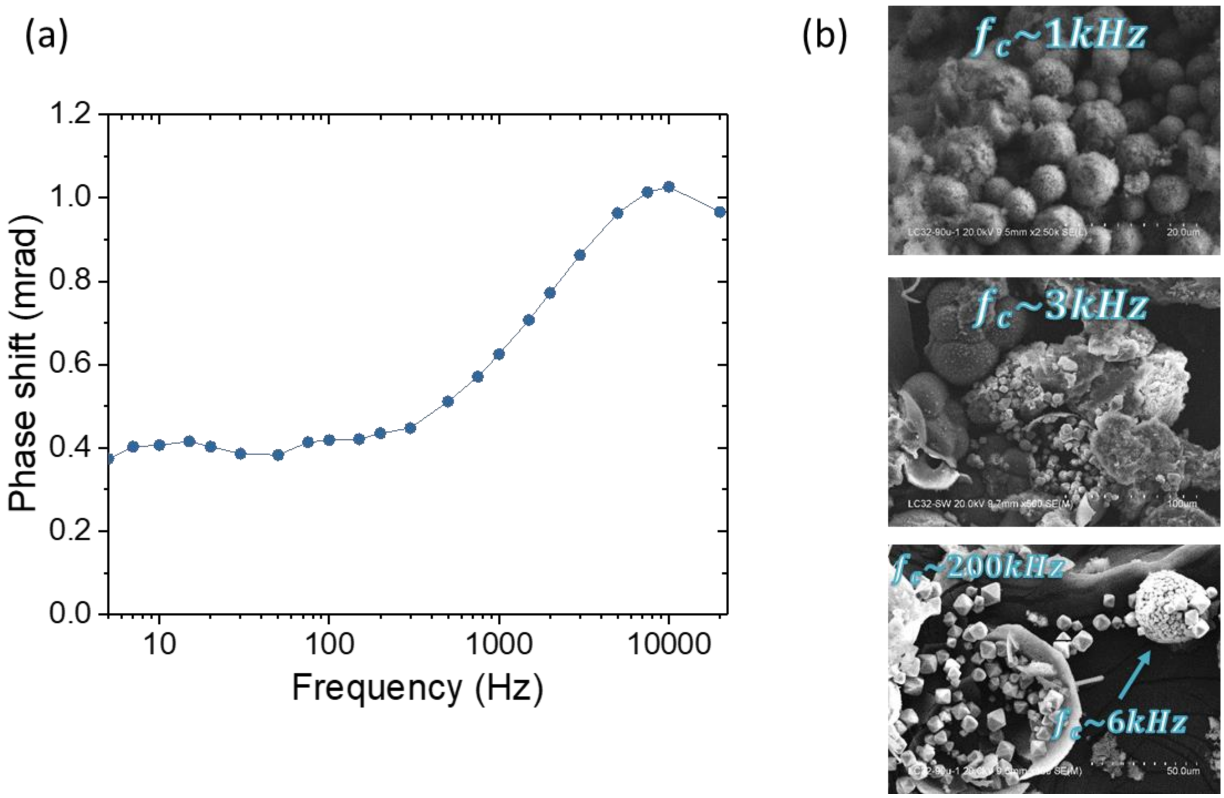


**Fig. S7.** (a) The induced polarization result with MD81-LC32 sample after sieving process; (b) Representative SEM images with euhedral pyrites and framboidal pyrites along with the predicted $\boldsymbol{f_c}$ labeled in the corresponding images

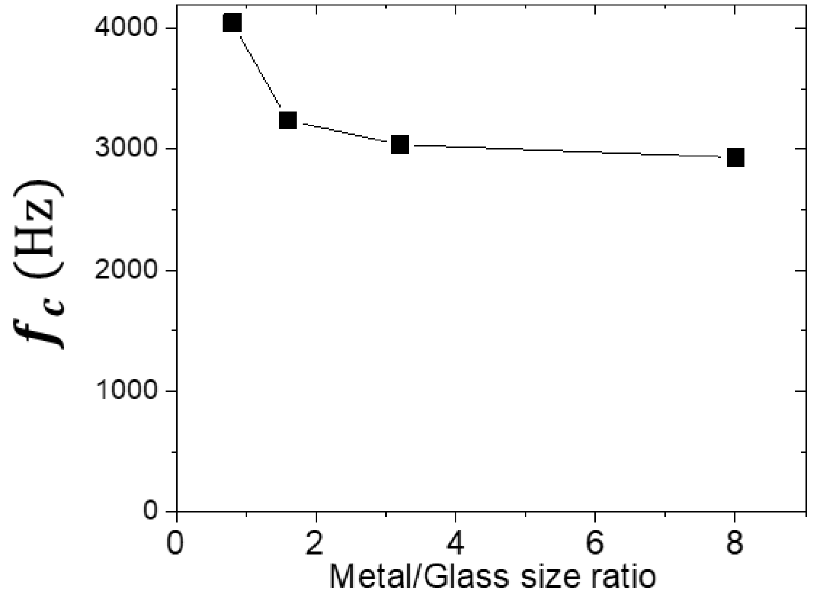


**Fig. S8.** Measured characteristic frequency changes respect to $a/a_g$. Tested in 3 wt% NaCl with 1.6 mm diameter 316 Stainless Steel spheres in monodispersed glass bead pack with different sizes of glass spheres.

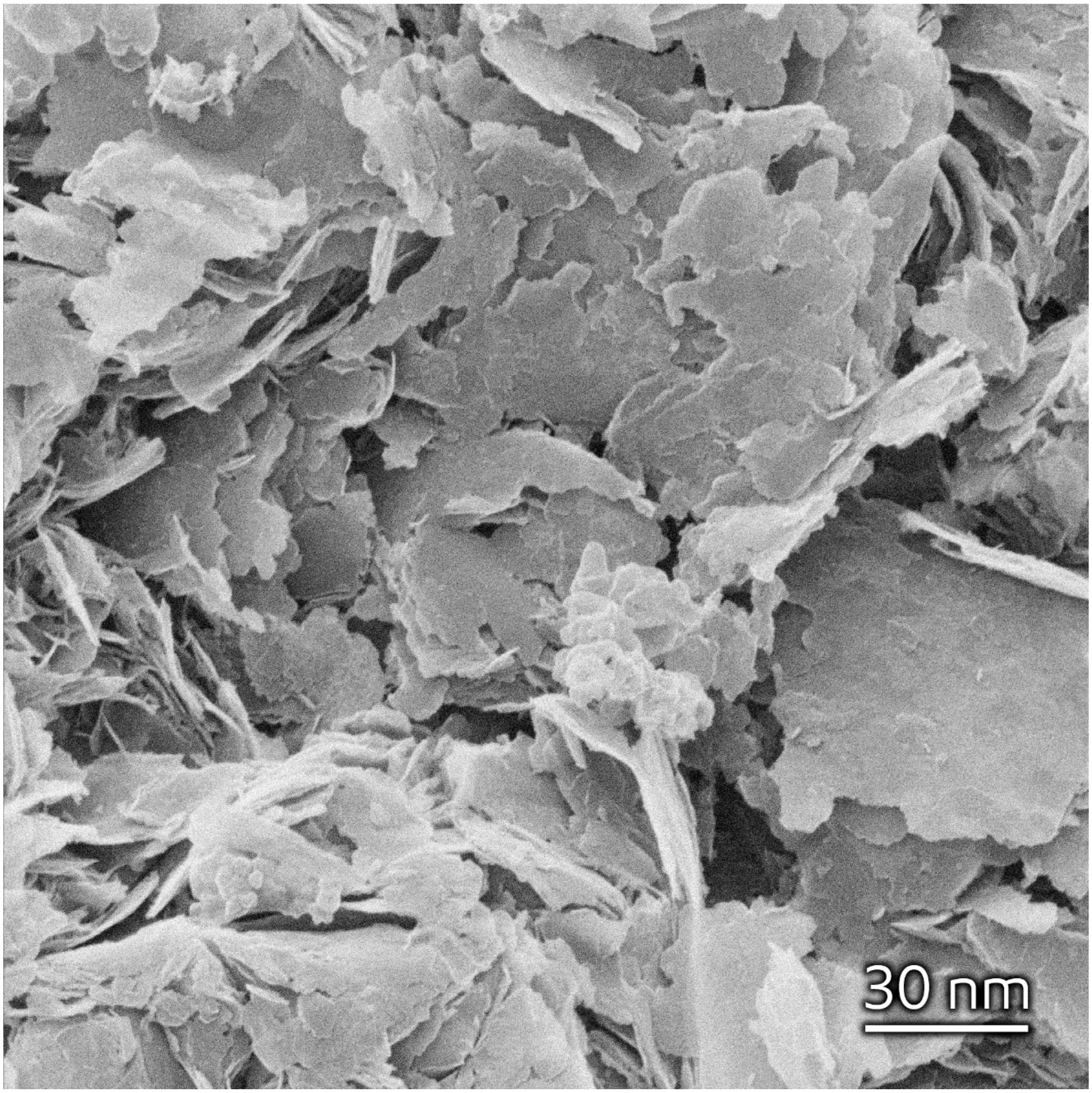


**Fig. S9.** Higher-resolution Helium ion microscopy image of nano-porous FeS layer on carbon steel beads after bacteria-induced corrosion.

## Supplementary Table

| Conducting Materials | Diameter or size (mm) | $V_{cond}$ | [NaCl] w/w | Effective Induced Polarization Parameters in glass bead pack | | | |
|---|---|---|---|---|---|---|---|
| | | | | $f_c\ (Hz)$ | $\phi_c (rad)$ | $\sigma_0\ (S/m)$ | $\sigma_\infty (S/m)$ |
| Gold Coated Spheres | 4 | 7.6% | 3% | 125 | 0.146 | 1.15 | 1.55 |
| Gold Coated Spheres | 4 | 7.6% | 0.30% | 16.6 | 0.147 | 0.141 | 0.191 |
| Gold Coated Spheres | 4 | 7.6% | 0.03% | 2 | 0.146 | 0.0148 | 0.0198 |
| Gold Coated Spheres | 2 | 7.0% | 3% | 287 | 0.138 | 1.01 | 1.33 |
| Gold Coated Spheres | 1 | 4.9% | 3% | 595 | 0.088 | 1.19 | 1.42 |
| | | 0.0% | | | | | |
| 316 Stainless Steel Spheres | 6.4 | 3.2% | 3% | 1363 | 0.065 | 1.29 | 1.47 |
| 316 Stainless Steel Spheres | 3.2 | 3.2% | 3% | 2704 | 0.068 | 1.2 | 1.38 |
| 316 Stainless Steel Spheres | 3.2 | 3.2% | 0.30% | 329 | 0.069 | 0.151 | 0.173 |
| 316 Stainless Steel Spheres | 3.2 | 3.2% | 0.03% | 30.5 | 0.069 | 0.0163 | 0.0187 |
| 316 Stainless Steel Spheres | 1.6 | 3.5% | 3% | 5162 | 0.071 | 1.27 | 1.45 |
| | | 0.0% | | | | | |
| Pyrite Cubes | 16 and 18 | 23.0% | 15% | 327 | 0.364 | 3.51 | 7.48 |
| Pyrite Cubes | 16 and 18 | 23.0% | 3% | 77.6 | 0.356 | 0.81 | 1.72 |
| Pyrite Cubes | 16 and 18 | 23.0% | 0.30% | 7.89 | 0.365 | 0.101 | 0.22 |
| Pyrite Cubes | 16 and 18 | 23.0% | 0.03% | 0.84 | 0.356 | 0.0114 | 0.0238 |
| Pyrite Cube | 16 | 9.4% | 3% | 79 | 0.165 | 1.06 | 1.5 |
| | | | | | | | |
| 304 Stainless Steel Spheres | 6.4 | 4.4% | 3% | 710 | 0.089 | 1.19 | 1.43 |
| 304 Stainless Steel Spheres | 6.4 | 4.4% | 0.30% | 53.5 | 0.087 | 0.141 | 0.168 |
| 304 Stainless Steel Spheres | 6.4 | 4.4% | 0.03% | 6.3 | 0.088 | 0.0163 | 0.0197 |
| | | 0.0% | | | | | |
| Platinum Coated Spheres | 6.4 | 2.9% | 3% | 57.9 | 0.063 | 1.32 | 1.5 |
| Platinum Coated Spheres | 6.4 | 2.9% | 0.30% | 5.7 | 0.0634 | 0.154 | 0.175 |
| Platinum Coated Spheres | 6.4 | 2.2% | 0.03% | 0.567 | 0.0468 | 0.0181 | 0.0196 |

**Table. S1.** Key detail for experimental systems with monodispersed conductive inclusions, and key induced polarization parameters after theoretical fitting with Equation S1.
